# SR-A1 cooperates with TLR4-NF-κB signaling to drive macrophage TGF-β1 expression and Treg induction during schistosome infection

**DOI:** 10.1371/journal.ppat.1014732

**Published:** 2026-10-30

**Authors:** Junyao Shen, Ziling Wang, Chuanhong Huang, Weijie Xue, Yu Wu, Tianyu Fan, Yuanyuan Miao, Chenxu Mao, Jiaojiao Yu, Yalin Li, Lei Xu, Jifeng Zhu, Xiaojun Chen, Yang Dai, Zhigang Lei, Sha Zhou, Chuan Su

**Affiliations:** 1 Key Laboratory for Pathogen Infection and Control of Jiangsu Province, Department of Pathogen Biology and Immunology, School of Basic Medical Sciences, National Vaccine Innovation Platform of Nanjing Medical University, State Key Laboratory of Reproductive Medicine and Offspring Health, Nanjing Medical University, Nanjing, Jiangsu, China; 2 Department of Immunology, School of Medicine, Nanjing University of Chinese Medicine, Nanjing, Jiangsu, China; 3 National Health Commission Key Laboratory of Parasitic Disease Control and Prevention, Jiangsu Provincial Key Laboratory on Parasite and Vector Control Technology, Jiangsu Institute of Parasitic Diseases, Wuxi, Jiangsu, China; 4 Key Laboratory for Pathogen Infection and Control of Jiangsu Province, Department of Pathogen Biology and Immunology, School of Basic Medical Sciences, Lianyungang Medical-Education Innovation and Research Center, Nanjing Medical University, Nanjing, Jiangsu, China; University of Notre Dame, UNITED STATES OF AMERICA

## Abstract

Helminths induce regulatory T cells (Tregs) to establish chronic infections, promoting long-term persistence of both parasites and hosts. TGF-β1 is essential for Treg induction by initiating Foxp3 expression. Helminth infections are typically associated with high TGF-β1 levels, yet the mechanisms by which helminths drive its production are not fully understood. Here, we uncover a mechanism whereby the scavenger receptor class A1 (SR-A1) cooperates with TLR4 signaling to drive TGF-β1 production by macrophages. Mechanistically, schistosome egg antigens activate TLR4-p50 (NF-κB1) while concurrently engaging SR-A1 to upregulate c-Jun, thereby assembling c-Jun-p50 transcription complexes at the *Tgfb1* promoter and enhancing TGF-β1 expression, subsequently inducing Tregs and mitigating liver immunopathology in *Schistosoma japonicum*-infected mice. Thus, we identify a previously unrecognized host-parasite interaction wherein SR-A1-TLR4 cooperation enables helminths to manipulate macrophage transcriptional programs for TGF-β1 expression and Treg induction, identifying a potential therapeutic target in the host for inflammatory helminthiasis.

## Author summary

Helminth parasites have evolved sophisticated strategies to persist in their hosts for prolonged periods, largely by actively dampening immune responses. A key strategy involves inducing regulatory T cells (Tregs), a specialized subset of immune cells that maintain tolerance and curb excessive inflammation. The cytokine TGF-β1 is essential for generating protective Tregs, yet the mechanisms by which parasitic worms stimulate their production remain poorly understood. This study unveils a previously unrecognized pattern recognition receptor crosstalk axis wherein helminth co-opts SR-A1-TLR4 synergy to orchestrate host tolerogenic programs during helminth infection. Mechanistically, schistosome egg antigens activate TLR4-p50 while engaging SR-A1 to upregulate c-Jun, forming c-Jun-p50 transcription complexes that enhance *Tgfb1* gene expression. This SR-A1-TLR4 axis induces regulatory T cells and mitigates liver immunopathology in infected mice. These findings illuminate a distinct host-parasite interface for governing immune homeostasis, identifying SR-A1 as a potential host-directed therapeutic target for modulating chronic parasitic immunopathology.

## Introduction

Helminth parasites are well-known for their strong immunoregulatory capacity, functioning to dampen the host’s anti-parasite immunity and protect the host against excessive immunopathology, thereby enabling the establishment of chronic infections and long-term persistence [1]. Although a wide variety of mechanisms are involved in helminth-induced immunoregulation, regulatory T cells (Tregs) play a functionally prominent role during helminth infections [2]. Of particular importance are mechanisms that drive *de novo* induction of peripheral Tregs by helminths [3].

Transforming growth factor-β1 (TGF-β1) is an essential factor for inducing *de novo* Foxp3 expression in naive CD4^+^ T cells and thus promoting the peripheral generation of CD4^+^CD25^+^Foxp3^+^ Tregs [4]. Helminth infections, as well as many other chronic infections (such as hepatitis B and C, tuberculosis, etc.), are typically associated with persistently and abundantly elevated TGF-β1 production, which has been tightly implicated in Treg induction [5,6]. However, the regulatory mechanisms coordinating TGF-β1 induction by helminths remain poorly understood.

Scavenger receptor class A1 (SR-A1; also known as MSR1 or CD204), a type of pattern recognition receptor, is predominantly expressed on tissue macrophages (Kupffer cells, etc.) and affects the activation status and function of macrophages during pathogen infection [7]. SR-A1 is upregulated on anti-inflammatory macrophages characterized by high expression of immunosuppressive cytokines and surface co-inhibitory molecules [7,8]. Therefore, SR-A1 might contribute to the immunosuppressive activity (e.g., TGF-β1 production) of anti-inflammatory macrophages. Despite lacking a discernible signaling motif within its cytoplasmic domain, SR-A1 cooperates with pattern recognition receptors (PRRs), such as TLR4 for *Escherichia coli* and TLR2 for *Staphylococcus aureus*, to recognize and phagocytose PAMPs and enhance innate immune responses [9]. Of note, we previously demonstrated that *Schistosoma japonicum* (*S. japonicum*) egg antigens (SEA) and their component heat shock protein 60 (SjHSP60), as a schistosomal egg-derived major contributor of Treg induction during infection, induce TGF-β1 expression via TLR4 signaling in macrophages [10]. However, whether SR-A1 has a critical regulatory role in TLR4-induced TGF-β1 expression and the underlying mechanisms remain to be resolved.

In this study, we demonstrated that SR-A1 promotes c-Jun expression to enable its interaction with p50 (NF-κB1), which is upregulated upon TLR4 activation, thereby facilitating the assembly of c-Jun-p50 transcription complexes for enhancing TGF-β1 expression in macrophages, which results in the induction of Tregs and the restriction of liver immunopathology in mice with *S. japonicum* infection.

## Results

### TGF-β1 augments Tregs and attenuates hepatic fibrosis in *S. japonicum*-infected mice

TGF-β1 is pivotal in the induction of hepatic Tregs to sustain local homeostasis [11]. Publicly available microarray data (GSE61376) revealed elevated hepatic *TGFB1* expression in schistosomiasis patients (Fig 1A). We observed a significant increase in TGF-β1 expression in the livers of *S. japonicum*-infected mice, as evidenced by RT-PCR and western blotting (Fig 1B and 1C). *S. japonicum* infection also induces pronounced elevation of circulating TGF-β1 in both humans and mice [10,12]. Elevated circulating active TGF-β1 was also confirmed in *S. japonicum*-infected mice (Fig 1D). Intraperitoneal administration of TGF-β1 further elevated both the proportion and absolute number of Tregs in the liver and spleen of infected mice (Fig 1E and 1F), whereas treatment with a TGF-β1-neutralizing antibody produced the opposite results (Fig 1G and 1H). Furthermore, TGF-β1 supplementation significantly attenuated hepatic fibrosis in *S. japonicum*-infected mice (Fig 1I); by contrast, TGF-β1-neutralizing antibody treatment exacerbated hepatic fibrosis (Fig 1J).

**Fig 1:**
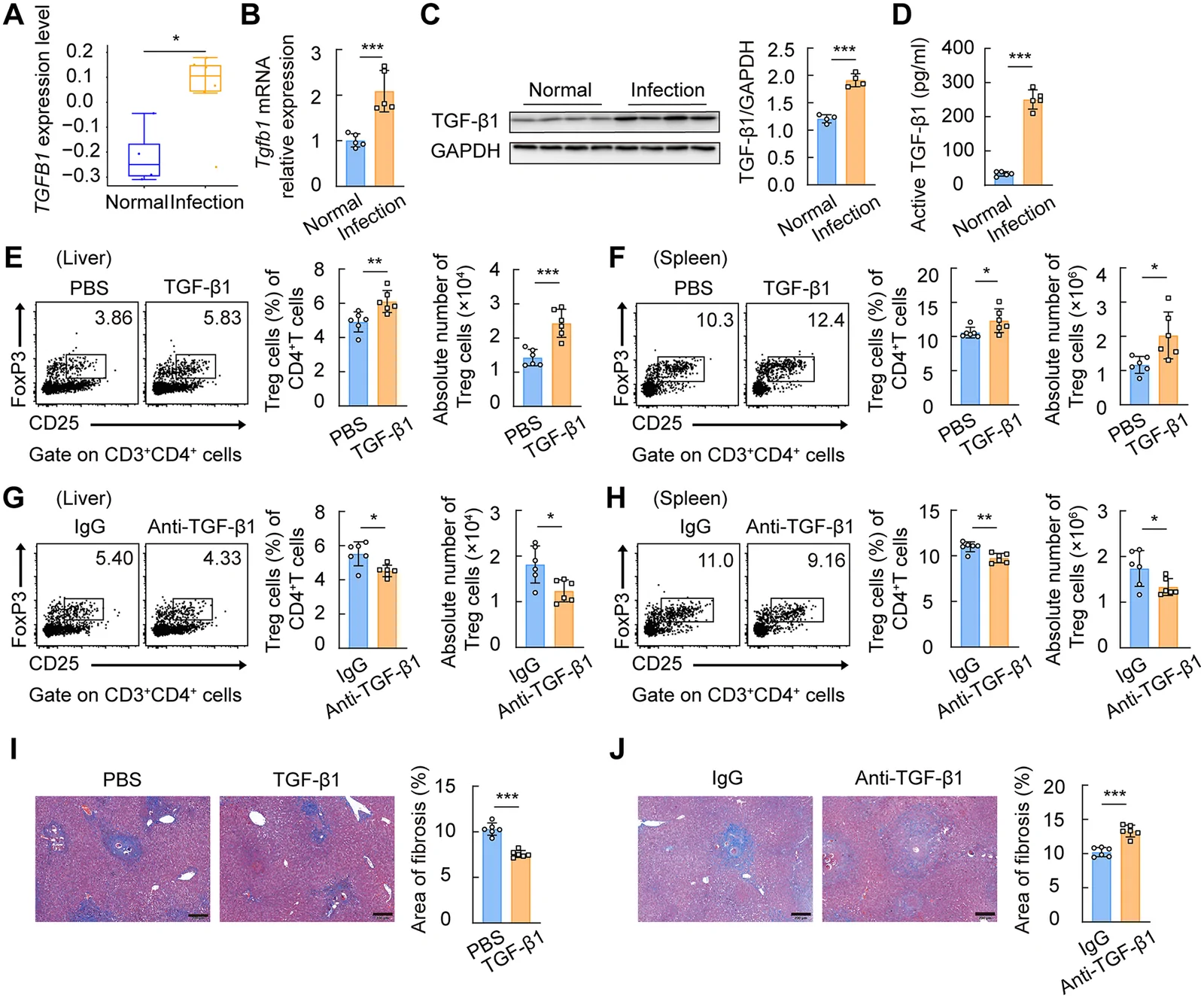
TGF-β1 augments Tregs and attenuates hepatic fibrosis in *S. japonicum*-infected mice. **(A)** Hepatic *TGFB1* expression in schistosomiasis patients based on the publicly available microarray dataset (GSE61376). **(B and C)** RT-PCR analysis of hepatic *Tgfb1* mRNA expression **(B)**, and Western blotting analysis of hepatic TGF-β1 levels **(C)**, in mice infected with *S. japonicum*. **(D)** Active TGF-β1 in serum was quantified by ELISA. **(E and F)** FCM analysis of CD4^+^CD25^+^Foxp3^+^ Tregs in the liver **(E)** and spleen **(F)** of *S. japonicum*-infected mice treated with PBS or TGF-β1. **(G and H)** FCM analysis of CD4^+^CD25^+^Foxp3^+^ Tregs in the liver **(G)** and spleen **(H)** of *S. japonicum*-infected mice treated with anti-TGF-β1 or isotype antibody. **(I and J)** Representative Masson staining of liver sections from *S. japonicum*-infected mice treated with PBS/TGF-β1 or isotype antibody/anti-TGF-β1. Scale bar: 200 µm. Liver fibrosis was quantified as the Masson-positive area (% area). Bar graphs show mean ± SD. Each dot represents an individual sample, and the number of dots in each group corresponds to its sample size (n). Comparing the means of two groups was performed using Student’s *t*-test (n ≥ 5) or Welch’s *t*-test (n < 5). *P < 0.05, **P < 0.01, ***P < 0.001.

### Macrophages are the primary cellular source of TGF-β1 in the liver of *S. japonicum*-infected mice

We next re-analyzed published single-cell RNA sequencing (scRNA-seq) results to identify the primary cellular source of TGF-β1 in the liver of *S. japonicum*-infected mice [13]. Results identified macrophage-predominant expression of TGF-β1 (Fig 2A-2C). Flow cytometric results showed that CD45^+^ hematopoietic cells were the main contributors to TGF-β1 expression in the liver of *S. japonicum*-infected mice (Fig 2D). Further analysis revealed that macrophages (CD11b^+^F4/80^+^SiglecF^−^) were the predominant source of TGF-β1 among CD45^+^ hematopoietic cells of the liver of *S. japonicum*-infected mice (Fig 2E). These results collectively indicate that macrophages contribute predominantly to increased TGF-β1 expression in the liver of *S. japonicum*-infected mice.

**Fig 2:**
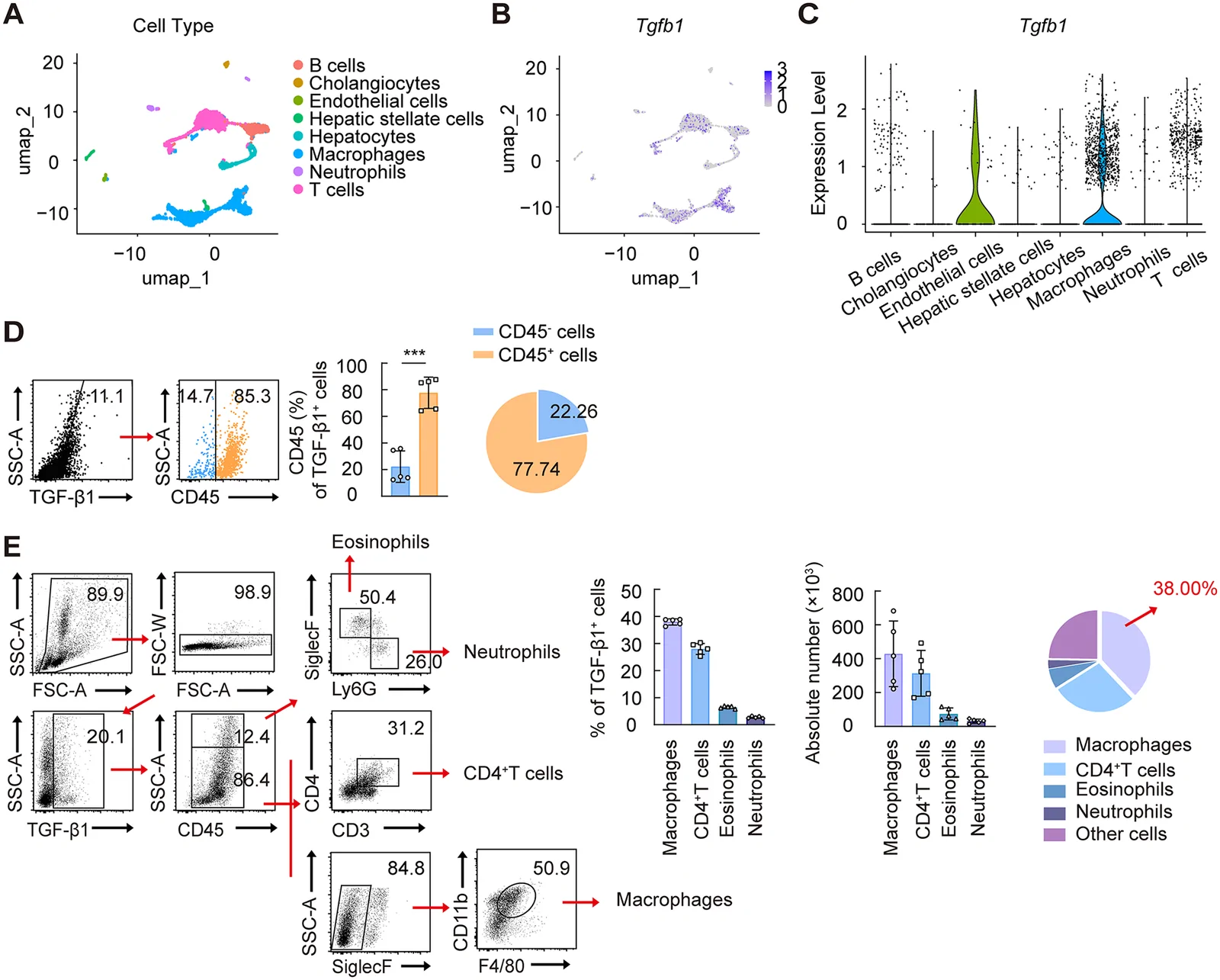
Macrophages are the primary cellular source of TGF-β1 in the liver of *S. japonicum*-infected mice. **(A-C)** ScRNA-seq analysis of *Tgfb1* expression in major cell populations of the liver from *S. japonicum*-infected mice, visualized using UMAP clustering (GSE220286). UMAP showing liver cell clusters **(A)**; *Tgfb1* expression mapped onto the clusters **(B)**; Violin plot showing *Tgfb1* expression across the liver cell clusters **(C)**. **(D)** FCM analysis of CD45^−^TGF-β1^+^ and CD45^+^TGF-β1^+^ cells in the liver of *S. japonicum*-infected mice. **(E)** FCM analysis of TGF-β1 expression in F4/80^+^CD11b^+^Siglec-F^−^ macrophages, CD11b^+^Siglec-F^+^ eosinophils, Ly6G^+^CD11b^+^ neutrophils, and CD3^+^CD4^+^ T cells. Representative FCM dot plots and quantification are shown. Bar and pie charts show the mean proportion of each indicated cell population. Bar graphs show mean ± SD. Each dot represents an individual sample, and the number of dots in each group corresponds to its sample size (n). Comparing the means of two groups was performed using Student’s *t*-test. ***P < 0.001.

### Deficiency of SR-A1 in macrophages results in reduced TGF-β1 expression and impaired Treg induction in *S. japonicum*-infected mice

Given that SR-A1 is predominantly expressed on tissue macrophages and upregulated in anti-inflammatory macrophages characterized by high expression of immunosuppressive cytokines including TGF-β1 [7,8], we hypothesized that SR-A1 regulates TGF-β1 production in hepatic macrophages during *S. japonicum* infection. Next, the scRNA-Seq analysis also showed that SR-A1 was predominantly expressed by macrophages in the livers of *S. japonicum*-infected mice (S1A and S1B Fig). Publicly available microarray datasets (GSE61376 and GSE14367) indicated that *S. japonicum* infection upregulated hepatic SR-A1 expression in both humans and mice (S1C and S1D Fig). Flow cytometry further confirmed an increase in SR-A1 expression in macrophages from the livers of *S. japonicum*-infected mice (S1E Fig). Following *S. japonicum* infection, both global *Msr1* knockout (*Msr1*^−/−^) and myeloid-specific *Msr1* knockout (*Msr1*^f/f^*Lyz2*^cre^) mice exhibited reduced TGF-β1 expression levels in the liver, as detected by RT-PCR and western blotting (Fig 3A and 3B). Consistently, active TGF-β1 levels in serum were significantly lower in *S. japonicum*-infected *Msr1*^−/−^ and *Msr1*^f/f^*Lyz2*^cre^ mice compared to control mice (Fig 3C).

**Fig 3:**
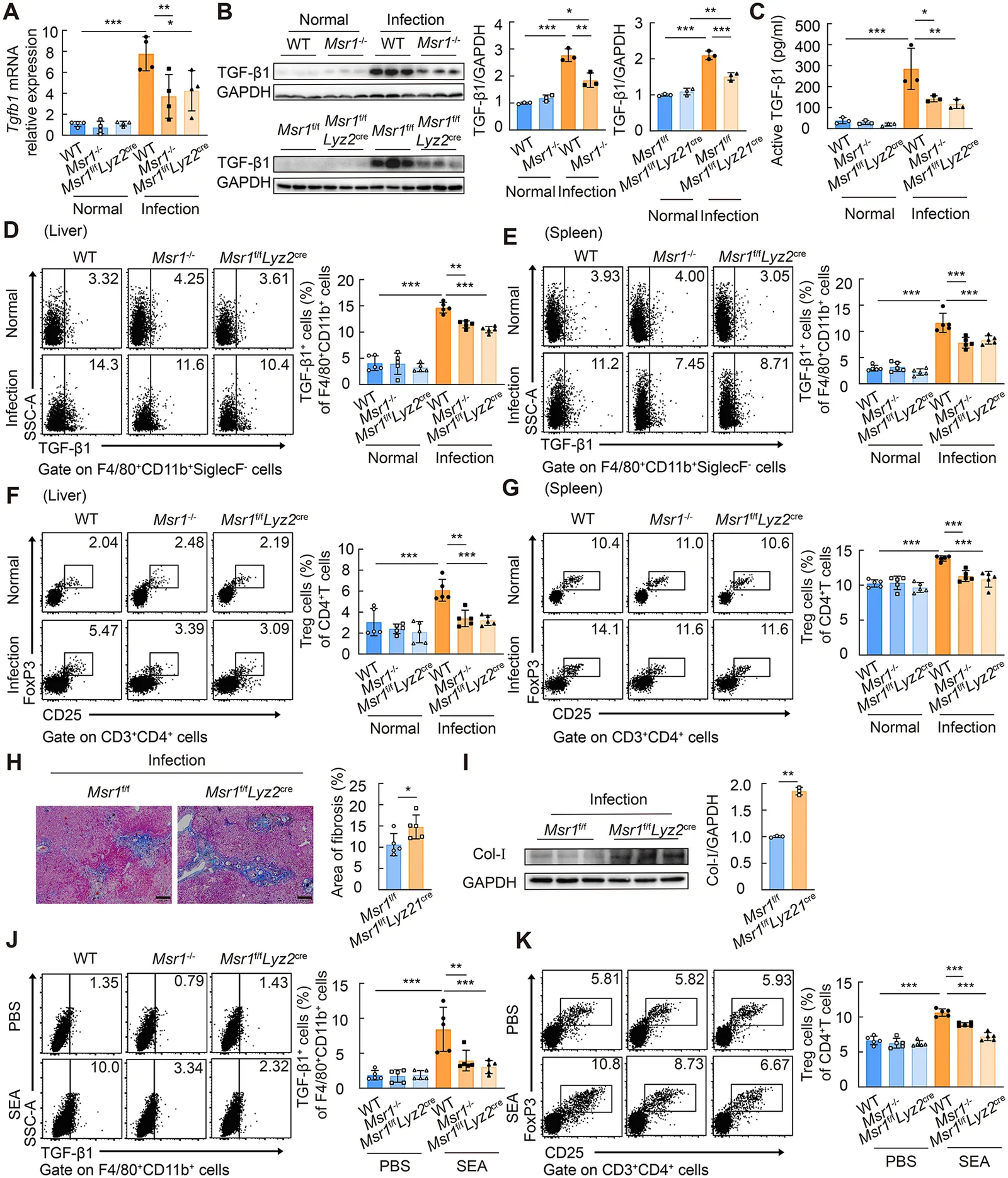
Deficiency of SR-A1 in macrophages results in reduced TGF-β1 expression and impaired Treg induction in *S. japonicum*-infected mice. **(A and B)** Hepatic *Tgfb1* mRNA and protein levels in wild-type (WT), *Msr1*^−/−^, and *Msr1*^f/f^*Lyz2*^cre^ mice with or without *S. japonicum* infection were quantified by RT-PCR **(A)** and Western blotting **(B)**, respectively. **(C)** Active TGF-β1 in serum was quantified by ELISA. **(D and E)** FCM analysis of TGF-β1 expression in F4/80^+^CD11b^+^Siglec-F^−^ macrophages from the liver **(D)** and spleen **(E)** of WT, *Msr1*^−/−^, and *Msr1*^f/f^*Lyz2*^cre^ mice with or without *S. japonicum* infection. **(F and G)** FCM analysis of CD4^+^CD25^+^Foxp3^+^ Tregs in the liver **(F)** and spleen **(G)** of WT, *Msr1*^−/−^, and *Msr1*^f/f^*Lyz2*^cre^ mice with or without *S. japonicum* infection. **(H)** Representative Masson staining of liver sections of *Msr1*^f/f^ and *Msr1*^f/f^*Lyz2*^cre^ mice infected with *S. japonicum*. Scale bar: 200 µm. Liver fibrosis was quantified as the Masson-positive area (% area). **(I)** Western blotting analysis and quantification of Col-I in the liver. **(J)** BMDMs from WT, *Msr1*^−/−^, and *Msr1*^f/f^*Lyz2*^cre^ mice were stimulated with SEA (10 μg/mL) or PBS for 48 hours. TGF-β1 expression in BMDMs was then analyzed by FCM. **(K)** Splenocytes from WT, *Msr1*^−/−^, and *Msr1*^f/f^*Lyz2*^cre^ mice were stimulated with SEA (10 μg/mL) or PBS for 48 hours. CD4^+^CD25^+^Foxp3^+^ Tregs were then analyzed by FCM. Bar graphs show the mean proportion of each indicated cell population. The data are expressed as the mean ± SD. Each dot represents an individual sample, and the number of dots in each group corresponds to its sample size (n). Comparing the means of two groups was performed using Student’s *t*-test (n ≥ 5) or Welch’s *t*-test (n < 5). The differences among three or more groups were analyzed with ANOVA. *P < 0.05, **P < 0.01, ***P < 0.001, ns indicates not significant.

In both *Msr1*^−/−^ and *Msr1*^f/f^*Lyz2*^cre^ mice with *S. japonicum* infection, the numbers of hepatic and splenic macrophages were comparable to those in the control groups (S2A and S2B Fig), indicating that the reduced hepatic TGF-β1 expression in these mice was not caused by a decrease in macrophage numbers. Flow cytometry analysis revealed a significant reduction in TGF-β1 expression in macrophages from the livers and spleens of *Msr1*^−/−^ or *Msr1*^f/f^*Lyz2*^cre^ mice infected with *S. japonicum*, compared to their wild-type (WT) controls (Fig 3D and 3E). Consistently, a reduction in Treg proportion was also observed in the livers and spleens of *Msr1*^−/−^ and *Msr1*^f/f^*Lyz2*^cre^ mice with *S. japonicum* infection (Fig 3F and 3G). Moreover, while IL-10 has also been implicated in Treg expansion [14,15], hepatic IL-10 levels remained unaffected in both *Msr1*^−/−^ and *Msr1*^f/f^*Lyz2*^cre^ mice infected with *S. japonicum* (S3A and S3B Fig). Importantly, *Msr1*^f/f^*Lyz2*^cre^ mice infected with *S. japonicum* exhibited more severe hepatic fibrosis compared to *Msr1*^f/f^ mice, as determined by Masson staining and Western blotting analysis of type I collagen (Col-I; Fig 3H and 3I), concomitant with elevated hepatic IL-13 levels indicative of enhanced type 2 immune responses in the liver (S3A and S3B Fig). SEA are the primary stimuli driving Treg induction during *S. japonicum* infection [16]. We thus conducted *in vitro* experiments using SEA and bone marrow-derived macrophages (BMDMs). First, we confirmed that SEA stimulation induced TGF-β1 expression in BMDMs, but this response was significantly diminished in SR-A1-deficient macrophages (Fig 3J). Accordingly, SEA-induced Treg generation was impaired in splenocytes isolated from *Msr1*^−/−^ or *Msr1*^f/f^*Lyz2*^cre^ mice (Fig 3K).

Collectively, these results demonstrate that SR-A1 deficiency in macrophages leads to reduced TGF-β1 expression and impaired Treg induction during *S. japonicum* infection.

### SR-A1 enhances SjHSP60-p50-mediated TGF-β1 expression in macrophages

Membrane SR-A1 lacks a discernible signaling motif in its cytoplasmic domain and typically interacts with other signaling pathways through association with membrane receptors or cytoplasmic components [8,17]. This raises the interesting question of how SR-A1 modulates TGF-β1 expression in macrophages. We then performed RNA-sequencing (RNA-seq) to compare the transcriptomes of macrophages from the livers of *Msr1*^f/f^ mice and *Msr1*^f/f^*Lyz2*^cre^ mice infected with *S. japonicum*. RNA-seq identified 66 up-regulated and 110 down-regulated differentially expressed genes (DEGs) (Fig 4A). The NF-κB signaling pathway exhibited the highest DEG enrichment in the environmental information processing KEGG module (Fig 4B).

**Fig 4:**
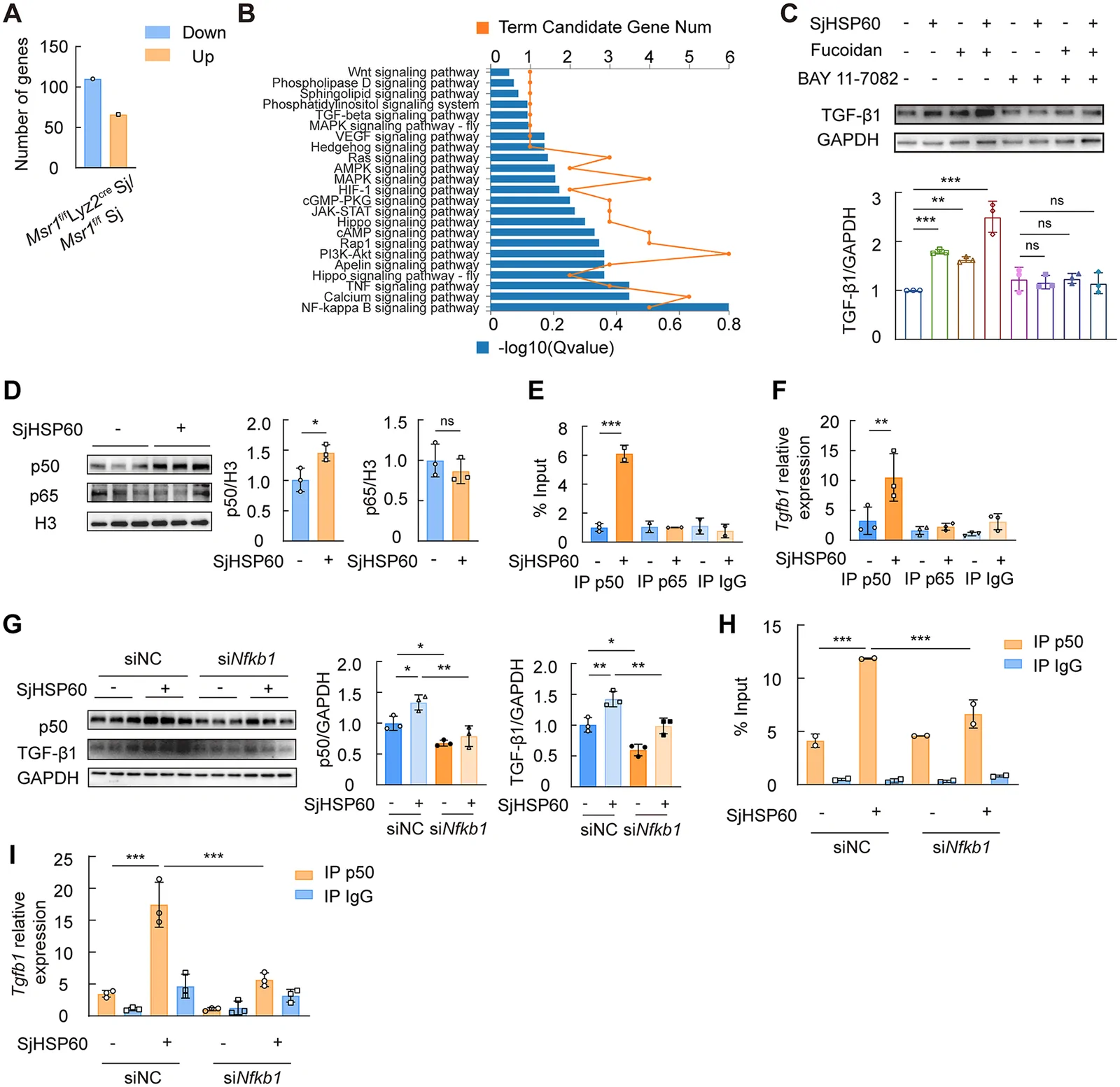
SR-A1 enhances SjHSP60-p50-mediated TGF-β1 expression in macrophages. **(A)** Number of differentially expressed genes in hepatic macrophages between *Msr1*^f/f^ and *Msr1*^f/f^*Lyz2*^cre^ mice. DEGs were identified with the thresholds of |log_2_FC| > 0.6 and adjusted *P*-value < 0.1. **(B)** KEGG pathway enrichment analysis of differentially expressed genes in hepatic macrophages from *Msr1*^f/f^ and *Msr1*^f/f^*Lyz2*^cre^ mice. **(C)** RAW264.7 macrophages were cultured with SjHSP60 (0.5 μg/mL) and/or fucoidan (100 μg/mL) for 48 hours, with or without BAY 11–7082 (an inhibitor of NF-κB; 10 μM). TGF-β1 expression in macrophages was determined by Western blotting. **(D-F)** RAW264.7 macrophages were cultured with SjHSP60 (0.5 μg/mL) for 48 hours. Nuclear fractions were extracted and subjected to Western blotting analysis of p50 and p65 **(D)**. A ChIP assay was performed to detect the association of p50 and p65 with the TGF-β1 promoter by qPCR **(E)**. The qPCR data were calculated as a percentage of the input sample. CUT&Tag assay was performed using anti-p50, anti-p65, or rabbit IgG, followed by qPCR **(F)**. **(G-I)** RAW264.7 macrophages transfected with *Nfkb1*/*p105* siRNA were stimulated with or without SjHSP60 (0.5 μg/mL) for 48 hours. TGF-β1 and p50 expression in macrophages was determined by Western blotting **(G)**. A ChIP assay was performed to detect the association of p50 with the *Tgfb1* promoter by qPCR **(H)**. CUT&Tag assay was performed using anti-p50 or rabbit IgG, followed by q-PCR **(I)**. Bar graphs show mean ± SD. Each dot represents an individual sample, and the number of dots in each group corresponds to its sample size (n). Comparing the means of two groups was performed using Welch’s *t*-tes*t* (n < 5). The differences among three or more groups were analyzed with ANOVA. *P < 0.05, **P < 0.01, ***P < 0.001, ns indicates not significant.

Despite lacking intrinsic signaling motifs, SR-A1 specifically partners with TLR4 for *Escherichia coli* phagocytosis [9]. In our prior study, we demonstrated that SjHSP60, a stress protein enriched in SEA, is primarily responsible for SEA-triggered TGF-β1 production in macrophages through the TLR4 pathway, thereby driving Treg induction [18]. Schistosome antigens are rich in ligands for both TLRs and SR-A1 [19]. We therefore postulated that schistosome antigens potentially orchestrate the cooperation between SR-A1 and TLR4.

SjHSP60 is a natural ligand for TLR4, yet no experimental evidence supports its interaction with SR-A1 to date. The SjHSP60-SR-A1 interaction was not supported by structural modeling based on AlphaFold. The most probable binding model shows pLDDT = 58.2, pTM = 0.488, and ipTM = 0.172, indicating that these two proteins are unlikely to form a stable complex (S4 Fig). *In vitro* experiments demonstrated that inhibition of TLR4 signaling with TAK-242 completely abrogated SjHSP60-induced TGF-β1 expression in macrophages (S5A Fig), thereby precluding SR-A1 involvement in this pathway. Fucoidan, a general ligand of the scavenger receptor, was typically used to trigger SR-A1 [8]. Importantly, fucoidan alone failed to induce TGF-β1 expression in SR-A1-deficient macrophages, indicating that, unlike SjHSP60, fucoidan utilizes SR-A1 but not TLR4 to induce TGF-β1 expression (S5B Fig).

By the combined use of fucoidan and SjHSP60, we next investigated whether SR-A1 cooperates with TLR4 to activate NF-κB signaling to induce TGF-β1 expression. Fucoidan alone only moderately increased TGF-β1 expression in RAW264.7 macrophages, but significantly enhanced SjHSP60-induced TGF-β1 expression (Fig 4C). Moreover, the NF-κB inhibitor BAY 11–7082 almost completely abolished both SjHSP60-induced and fucoidan-enhanced TGF-β1 expression (Fig 4C). Importantly, the supernatant derived from BMDMs stimulated with SjHSP60 and fucoidan exhibited elevated active TGF-β1 levels and significantly promoted Treg generation in splenocytes in a TGF-β1 signaling-dependent manner (S6B Fig). These results indicate that SR-A1 facilitates SjHSP60-activated NF-κB signaling to enhance TGF-β1 production in macrophages.

Next to explore how SR-A1 manipulates NF-kB signaling to enhance SjHSP60-induced TGF-β1 expression, we first needed to clarify how SjHSP60 activates NF-κB signaling to induce TGF-β1 expression in macrophages. NF-κB activation is mediated by the IκB kinase (IKK) complex, consisting of two catalytic subunits, IKKα and IKKβ, and the regulatory subunit IKK-γ. IKKα and IKKβ are associated with non-canonical and canonical NF-κB activation, respectively [20]. Our results demonstrated that SjHSP60 specifically upregulated IKKα expression, but not IKKβ, in macrophages, as confirmed by Western blotting analysis (S7 Fig).

IKK directly phosphorylates the inhibitory molecule IκBα, leading to the activation of NF-κB subunits, which then translocate to the nucleus as homo- or heterodimers to initiate transcription of target genes [20]. Next, NF-κB activation in SjHSP60-stimulated macrophages was characterized by assessing the nuclear translocation of canonical NF-κB subunits, p50 and p65. Compared to untreated macrophages, SjHSP60-treated macrophages exhibited significantly higher nuclear p50 levels, while nuclear p65 levels remained unchanged (Fig 4D).

To explore whether p50 interacts with the *Tgfb1* gene promoter, we performed ChIP assays on SjHSP60-stimulated Mφ. Our results showed that SjHSP60 significantly enhanced p50 binding to the *Tgfb1* promoter, whereas p65 displayed no significant binding to the promoter (Fig 4E). The ChIP results were independently validated by CUT&Tag-qPCR (Fig 4F). To further investigate whether p50 binding to the TGF-β1 promoter is critical for TGF-β1 production, we used siRNA duplexes targeting *Nfkb1*, the precursor of p50, to specifically knock down p50 expression. The *Nfkb1* siRNA duplexes reduced p50 levels, resulting in decreased p50 binding to the *Tgfb1* promoter and reduced TGF-β1 expression in SjHSP60-stimulated macrophages (Fig 4G and 4H). CUT&Tag-qPCR assays confirmed that p50 occupancy at the *Tgfb1* promoter was markedly diminished upon *Nfkb1* silencing (Fig 4I). Together, these *in vitro* results indicate that SR-A1 enhances SjHSP60-p50-mediated expression of TGF-β1 in macrophages.

### P50 is essential for SR-A1 to promote macrophage TGF-β1 expression and Treg induction in *S. japonicum*-infected mice

Next, to further confirm the role of p50 in SR-A1-enhanced TGF-β1 production by macrophages *in vivo*, we constructed adeno-associated virus (AAV) vectors for F4/80 promoter-driven NF-κB1/p105 knockdown (miR30-based shRNA targeting *Nfkb1*, AAV-shNfkb1) in macrophages of *S. japonicum*-infected *Msr1*^f/f^ and *Msr1*^f/f^*Lyz2*^cre^ mice. The experimental design is outlined in Fig 5A. The efficiency of NF-κB1 knockdown in mouse liver and spleen macrophages was verified by flow cytometry (Fig 5B and 5C). SR-A1 ablation in *Msr1*^f/f^*Lyz2*^cre^ mice produced a minor but not significant impact on macrophage NF-κB1 expression (Fig 5B and 5C). Compared to *Msr1*^f/f^ mice, *Msr1*^f/f^*Lyz2*^cre^ mice infected with *S. japonicum* exhibited lower TGF-β1 expression in macrophages and fewer Tregs in the liver and spleen (Fig 5D–5G). NF-κB1 knockdown reduced macrophage TGF-β1 expression and Treg induction in *S. japonicum*-infected *Msr1*^f/f^ mice, though these effects were attenuated in *Msr1*^f/f^*Lyz2*^cre^ mice with *S. japonicum* infection (Fig 5D–5G). Consistently, NF-κB1 knockdown exacerbated liver fibrosis in *S. japonicum*-infected *Msr1*^f/f^ mice but had no obvious effect in *Msr1*^f/f^*Lyz2*^cre^ mice (Fig 5H and 5I). Collectively, these *in vivo* results indicate that p50 is essential for SR-A1 to promote macrophage TGF-β1 expression and Treg induction, resulting in alleviation of immunopathology in mice infected with *S. japonicum*.

**Fig 5:**
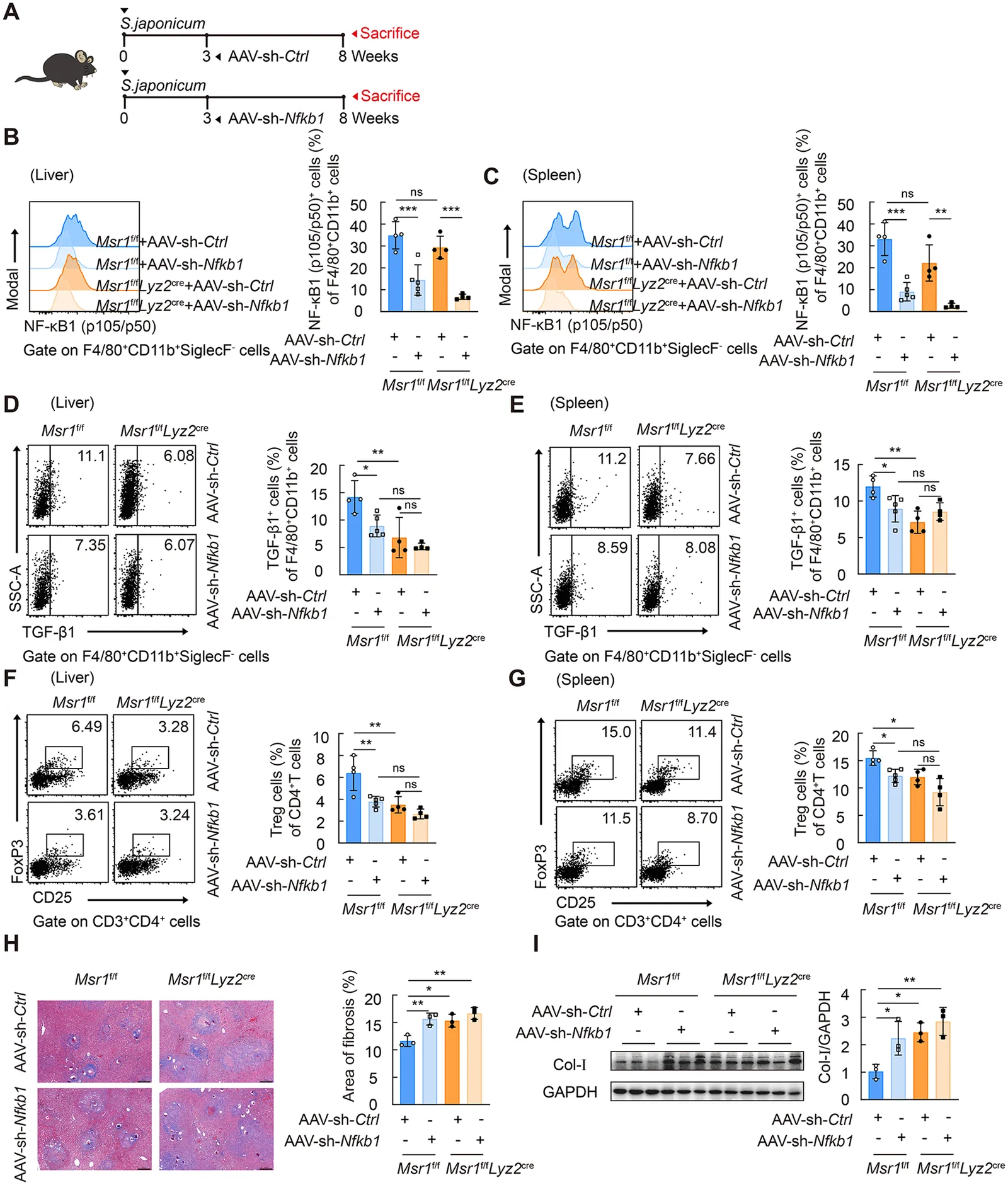
p50 is essential for SR-A1 to promote macrophage TGF-β1 expression and Treg induction in *S. japonicum*-infected mice. **(A)** Schematic of the experimental methods, showing the injection of AAV9 vectors (AAV-shNfkb1 or AAV-shCtrl) in *Msr1*^f/f^ and *Msr1*^f/f^*Lyz2*^cre^ mice infected with *S. japonicum*. **(B and C)** FCM analysis of NF-κB1 expression in hepatic **(B)** and splenic **(C)** macrophages from mice following AAV9 vector injection. **(D and E)** FCM analysis of TGF-β1 expression in hepatic **(D)** and splenic **(E)** macrophages from mice following AAV9 vector injection. **(F and G)** FCM analysis of CD4^+^CD25^+^Foxp3^+^ Tregs in liver **(F)** and spleen **(G)** from mice following AAV9 vector injection. **(H and I)** Representative Masson staining of liver sections of mice following AAV9 vector injection. Scale bar: 200 µm (**H**, left). Liver fibrosis was quantified as the Masson-positive area (**H**, right; % area). **(I)** Western blotting analysis and quantification of Col-I in the liver. Bar graphs show mean ± SD. Each dot represents an individual sample, and the number of dots in each group corresponds to its sample size (n). The differences among groups were analyzed with ANOVA. *P < 0.05, **P < 0.01, ***P < 0.001, ns indicates not significant.

### SR-A1 upregulates c-Jun to facilitate c-Jun-p50 complex formation and thereby enhances SjHSP60-induced TGF-β1 expression in macrophages

Due to the lack of transcriptional activity in p50 [21], we speculated that co-activators, which may be activated by SR-A1, are required in the transcription initiation complex. Studies of other diseases (e.g., leishmaniasis, asthma) in distinct cell types (e.g., T cells, monocytes) have identified AP-1 (c-Jun and c-Fos), HIF-1α, STAT3, and PPARδ as transcription activators involved in TGF-β1 transcription activation [22–25]. STRING database analysis identified the highest-confidence interaction between c-Jun and p50 (Score = 0.844; Fig 6A). Inter-molecular interactions were docked using the HDOCK server to a cartoon representation of the c-Jun-p50 complex (Fig 6B). Besides, JASPAR analysis revealed adjacent binding sites for p50 (nucleotides: 550–560) and c-Jun (nucleotides: 561–573) within the *Tgfb1* promoter (Fig 6C), further supporting their potential cooperation as a transcriptional complex to activate *Tgfb1* transcription. Reciprocal co-immunoprecipitation (Co-IP) experiments were performed in whole-cell and nuclear lysates, and physical interactions among them were shown (Fig 6D).

**Fig 6:**
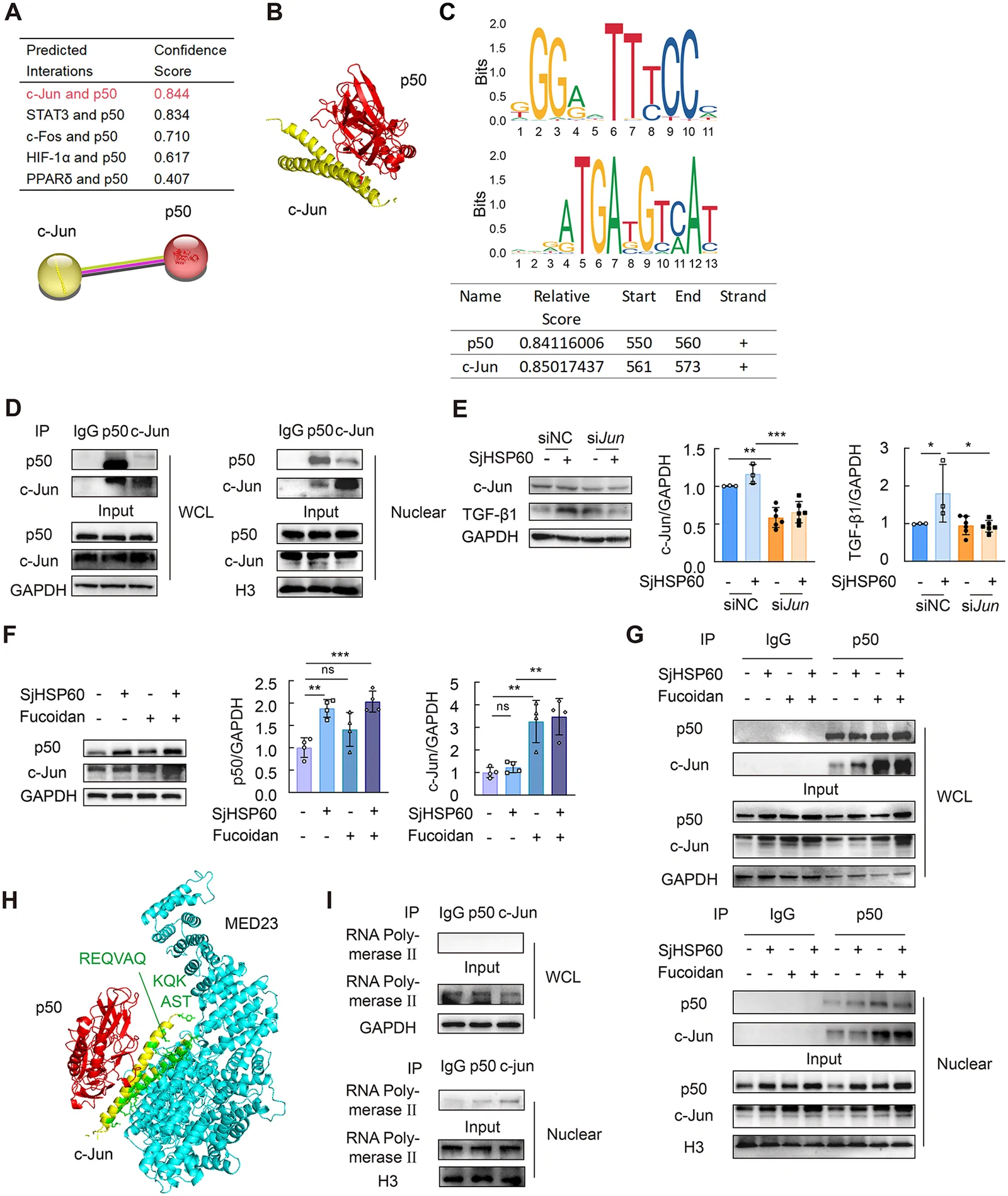
SR-A1 upregulates c-Jun to facilitate c-Jun-p50 complex formation, thereby enhancing SjHSP60-induced TGF-β1 expression in macrophages. **(A)** Protein interaction network generated using the STRING database (c-Jun, c-Fos, STAT3, PPARδ, HIF-1α, and p50). **(B)** The inter-molecular interaction between c-Jun (yellow) and p50 (red) was modeled using the HDOCK server (http://hdock.phys.hust.edu.cn/). **(C)** JASPAR prediction of the putative binding sites for p50 (nucleotides: 550–560) and c-Jun (nucleotides: 561–573) within the *Tgfb1* promoter. **(D)** Co-immunoprecipitation analysis of the interaction between c-Jun and p50 in whole-cell lysates (WCL) and nuclear extracts of RAW264.7 macrophages following stimulation with SjHSP60 (0.5 μg/mL) for 48 hours. **(E)** RAW264.7 macrophages transfected with *c-jun* siRNA were stimulated with or without SjHSP60 (0.5 μg/mL) for 48 hours. TGF-β1 expression in macrophages was determined by Western blotting. **(F)** RAW264.7 macrophages were cultured with SjHSP60 (0.5 μg/mL) and/or fucoidan (100 μg/mL) for 48 hours. Expression levels of p50 and c-Jun in macrophages were determined by Western blotting. **(G)** Co-immunoprecipitation analysis of the interaction between c-Jun and p50 in whole-cell lysates (WCL) and nuclear extracts of macrophages. **(H)** The inter-molecular interactions among c-Jun (yellow), p50 (red), and MED23 (blue) were modeled using the HDOCK server (http://hdock.phys.hust.edu.cn/). The basic leucine zipper (bZIP) domain of c-Jun (residues 279–315) is shown in green. **(I)** Co-immunoprecipitation analysis of interactions among c-Jun, p50, and RNA polymerase II in whole-cell lysates (WCL) and nuclear extracts of RAW264.7 macrophages following stimulation with SjHSP60 (0.5 μg/mL) for 48 hours. Bar graphs show mean ± SD. Each dot represents an individual sample, and the number of dots in each group corresponds to its sample size (n). The differences among groups were analyzed with ANOVA. *P < 0.05, **P < 0.01, ***P < 0.001.

We next determined whether c-Jun is critical for SjHSP60-induced expression of TGF-β1. Specific small interfering RNA (siRNA) was used to knock down c-Jun expression (Fig 6E). Results showed that c-Jun knockdown markedly abrogated SjHSP60-induced TGF-β1 in macrophages (Fig 6E).

Hepatic and splenic macrophages from *S. japonicum*-infected mice exhibited upregulated c-Jun expression compared with control mice (S8A and S8B Fig). *In vitro* results revealed that fucoidan treatment, which typically activates SR-A1, resulted in elevated c-Jun expression, but did not significantly alter p50 levels (Fig 6F). In contrast, SjHSP60 upregulated p50 levels without affecting c-Jun expression (Fig 6F). Of note, the combination of fucoidan and SjHSP60 concurrently increased c-Jun and p50 levels (Fig 6F) and enhanced c-Jun-p50 formation in the nucleus of macrophages (Fig 6G).

We further explored how c-Jun-p50 activates *Tgfb1* transcription. Mediator (MED) is an essential transcriptional co-activator that bridges gene-specific transcription factors and RNA polymerase II to facilitate assembly of the transcription complex at promoters [26]. MED23 serves as a critical coactivator of transcriptional programs induced by PAMPs (e.g., LPS) and heat shock, while also modulating the c-Jun response [27,28]. Computational docking using the HDOCK server predicted a stable binding configuration for the p50-c-Jun-MED23 complex, wherein MED23 interacts with c-Jun rather than p50 (Score = 0.948; Fig 6H). PyMOL interface analysis further predicted that MED23 primarily interacts with the basic-region leucine-Zipper domain of c-Jun (residues 279–315 shown in green; Fig 6H). Co-IP assays confirmed that the transcriptional complex comprising c-Jun, p50, and RNA polymerase II was predominantly localized to the nucleus (Fig 6I). Collectively, these results indicate that SR-A1 up-regulates c-Jun expression to promote the assembly of the p50-c-Jun-Med23-RNA polymerase II transcriptional complex, which enhances SjHSP60-induced TGF-β1 transcription and expression in macrophages.

### SR-A1-induced c-Jun depends on p50 to promote TGF-β1 expression and Treg induction in mice infected with *S. japonicum*

We next investigated *in vivo* whether macrophage SR-A1 promotes c-Jun expression to enhance TLR4-p50-induced TGF-β1 expression in *S. japonicum*-infected mice. An AAV vector expressing *Jun* under the control of the F4/80 promoter was constructed to induce macrophage-specific c-Jun overexpression in *Msr1*^f/f^ and *Msr1*^f/f^*Lyz2*^cre^ mice with *S. japonicum* infection (Fig 7A). Successful overexpression of c-Jun in hepatic and splenic macrophages was verified using FCM analysis (Fig 7B and 7C). As expected, macrophages from *Msr1*^f/f^*Lyz2*^cre^ mice displayed reduced c-Jun expression compared to *Msr1*^f/f^ mice (Fig 7B and 7C). Notably, we observed that c-Jun overexpression significantly rescued TGF-β1 expression in macrophages of *Msr1*^f/f^*Lyz2*^cre^ mice with *S. japonicum* infection (Fig 7D and 7E). However, these effects were reversed by p50-knockdown in macrophages (Fig 7D and 7E). Correspondingly, *Msr1*^f/f^*Lyz2*^cre^ mice rescued with c-Jun overexpression in macrophages showed increased hepatic Tregs and reduced liver fibrosis after *S. japonicum* infection (Fig 7F and 7G). However, all these effects of c-Jun overexpression were significantly reversed by p50-knockdown in macrophages (Fig 7F and 7G). These results indicate that SR-A1-induced c-Jun depends on TLR4-induced p50 to promote TGF-β1 expression and Treg induction in mice infected with *S. japonicum*.

**Fig 7:**
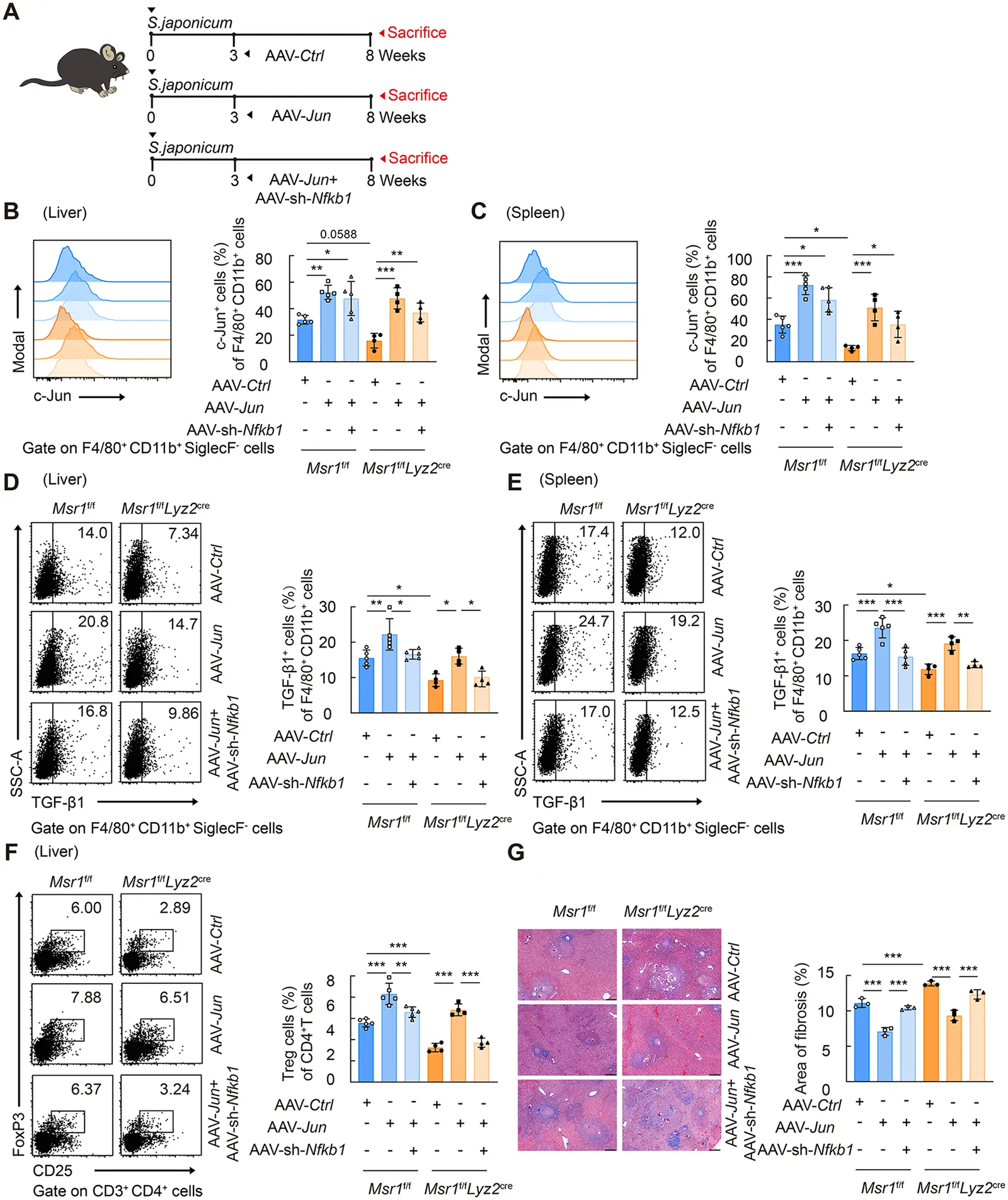
SR-A1-induced c-Jun depends on p50 to promote TGF-β1 expression and Treg induction in mice infected with *S. japonicum.* (A) Schematic of the experimental methods, showing the injection of AAV9 vectors (AAV-Ctrl, AAV-c-Jun, and/or AAV-shNfkb1) in *Msr1*^f/f^ and *Msr1*^f/f^*Lyz2*^cre^ mice infected with *S. japonicum*. (B and C) FCM analysis of c-Jun expression in hepatic **(B)** and splenic (C) macrophages from mice following AAV9 vector injection. (D and E) FCM analysis of TGF-β1 expression in hepatic (D) and splenic (E) macrophages from mice following AAV9 vector injection. (F) FCM analysis of CD4^+^CD25^+^Foxp3^+^ Tregs in the liver from mice following AAV9 vector injection. (G) Representative Masson staining of liver sections of mice following AAV9 vector injection. Scale bar: 200 µm. Liver fibrosis was quantified as the Masson-positive area (% area). Bar graphs show mean ± SD. Each dot represents an individual sample, and the number of dots in each group corresponds to its sample size (n). The differences among groups were analyzed with ANOVA. *P < 0.05, **P < 0.01, ***P < 0.001.

### Fucoidan promotes macrophage TGF-β1 expression and Treg induction in *S. japonicum*-infected mice dependent on both NF-κB and AP-1 signaling

Considering the progressive hepatic immunopathology and associated mortality in schistosomiasis patients, as well as the current lack of effective interventions, we thus evaluated whether treatment of *S. japonicum*-infected mice with the SR-A1 ligand fucoidan, a sulphated polysaccharide mainly isolated from edible brown seaweeds (marine algae) [29], could activate SR-A1 to promote macrophage TGF-β1 expression and Treg induction to restrain liver immunopathology. The experimental design is shown in S9 Fig. In line with our *in vitro* findings, fucoidan treatment significantly elevated c-Jun expression (Fig 8A and 8B) yet did not increase p50 expression in hepatic and splenic macrophages of *S. japonicum*-infected mice (Fig 8C and 8D). As expected, fucoidan treatment enhanced TGF-β1 expression in these macrophages of *S. japonicum*-infected mice (Fig 8E and 8F). Western blotting analysis also revealed a significant increase in hepatic TGF-β1 levels in *S. japonicum*-infected mice with fucoidan treatment (Fig 8G). Notably, inhibiting either NF-κB activation or AP-1 transcription activity with specific inhibitors (BAY 11–7082 and T5224, respectively) effectively blocked these *in vivo* effects (Fig 8E–8G). These results suggest that SR-A1-mediated promotion of TGF-β1 expression in macrophages relies on both NF-κB signaling and c-Jun activity during *S. japonicum* infection. The induction of Tregs in the liver and spleen of *S. japonicum*-infected mice yielded similar changes to those of TGF-β1 expression (Fig 8H and 8I). Concomitantly, fucoidan treatment significantly attenuated liver fibrosis in *S. japonicum*-infected mice, while either BAY 11–7082 or T5224 abolished these protective effects (Fig 8J and 8K). These *in vivo* data demonstrated that fucoidan upregulates c-Jun to enhance macrophage TGF-β1 expression and Treg induction, thereby alleviating hepatic pathology during schistosome infection.

**Fig 8:**
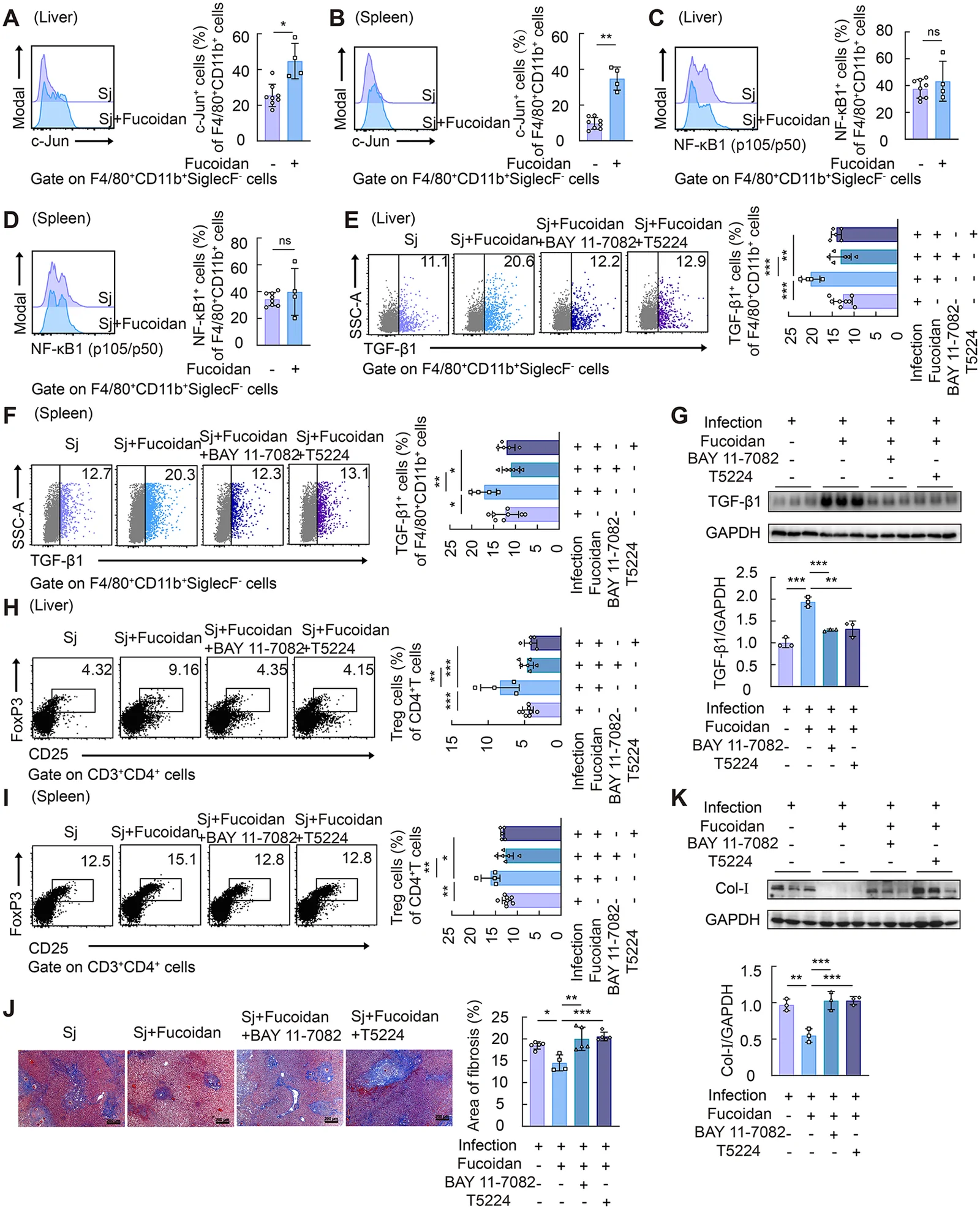
Fucoidan promotes macrophage TGF-β1 expression and Treg induction in *S. japonicum*-infected mice dependent on both NF-κB and AP-1 signaling. **(A-K)** FCM analysis of c-Jun **(A and B)** and p50 **(C and D)** expression in hepatic and splenic macrophages from *S. japonicum*-infected mice with fucoidan treatment. FCM analysis of TGF-β1 expression in hepatic **(E)** and splenic **(F)** macrophages from *S. japonicum*-infected mice following the above treatments. TGF-β1 expression in the liver of mice was determined by Western blotting **(G)**. FCM analysis of CD4^+^CD25^+^Foxp3^+^ Tregs in the liver **(H)** and spleen **(I)** of mice. Representative Masson staining of liver sections of mice. Scale bar: 200 µm (**J**, left). Liver fibrosis was quantified as the Masson-positive area (**J**, right; % area). Western blotting analysis and quantification of Col-I in the liver **(K)**. Each dot represents an individual sample, and the number of dots in each group corresponds to its sample size (n). Comparing the means of two groups was performed using Student’s *t*-test (n ≥ 5) or Welch’s *t*-test (n < 5). The differences among three or more groups were analyzed with ANOVA. *P < 0.05, **P < 0.01, ***P < 0.001, ns indicates not significant.

## Discussion

Mice with TGF-β1 deficiencies exhibit early postnatal lethality due to multiorgan inflammation [30]. As a key modulator of innate and adaptive immunity, TGF-β1 is essential for the maintenance of immune tolerance [31]. Persistent pathogens, especially long-lived and inherently immunetolerogenic helminth parasites, actively elicit high levels of TGF-β1 [3]. More importantly, TGF-β1 is essential for the peripheral differentiation of Tregs, which are necessary in mitigating immune pathology in nearly all studied parasite infections [32]. Helminths manipulate various PRRs, such as TLRs, to induce TGF-β1 production [33]. However, the precise regulatory mechanisms remain largely unelucidated. Herein, we demonstrated that activation of SR-A1, another innate PRR, boosts TGF-β1 expression in macrophages by upregulating c-Jun and forming more c-Jun-p50 transcription complexes, subsequently inducing Tregs and mitigating liver immunopathology during *S. japonicum* infection.

TGF-β1, ubiquitously expressed by multiple cell types, orchestrates multicellular responses to maintain homeostasis in health [31]. Although TGF-β1 is widely recognized as a canonical pro-fibrotic cytokine, its function is highly context-dependent [34]. In *S. japonicum* infection, our results demonstrated that host liver macrophages are driven to robustly express TGF-β1 and become the main source of TGF-β1 in the pathogenic liver microenvironment, with this cytokine primarily acting as an immunoregulator by inducing Tregs to dampen excessive granulomatous inflammation, thereby indirectly alleviating fibrosis. IL-13, as a pivotal type 2 immune effector molecule, is the dominant pro-fibrogenic cytokine for driving hepatic fibrosis in schistosomiasis [35,36]. As expected, we observed higher hepatic IL-13 levels in *Msr1^−/−^* and *Msr1*^f/f^*Lyz2*^cre^ mice infected with *S. japonicum*. Therefore, our findings provide mechanistic evidence that SR-A1-driven TGF-β1 production, which expands Tregs and operates hepatic protection, at least in part, by constraining type 2 immune responses and limiting IL-13-driven fibrogenesis.

Of note, TGF-β1, as a broadly immunosuppressive mediator, also has dramatic effects on macrophages [31]. Therefore, high TGF-β1 expression by macrophages during *S. japonicum* infection not only facilitates peripheral Treg induction but also triggers feedforward circuits to maintain macrophage-driven immunosuppression. Macrophages in chronically schistosome-infected liver exhibit immunosuppressive capacity akin to tumor-associated macrophages driven by tumor microenvironmental cues [37]. Elucidating how schistosomes motivate macrophages to express TGF-β1 is crucial for advancing our understanding of hepatic schistosomiasis and developing effective treatments.

TLRs not only trigger innate immune defense against pathogens but can also be modulated by helminth-derived molecules to induce a tolerogenic phenotype in innate immune cells secreting anti-inflammatory cytokines [38,39]. Of note, we previously demonstrated that both SEA and its essential component SjHSP60 induce TGF-β1 expression in macrophages through TLR4 signaling [10]. The diverse outcomes of TLR signaling imply that their function may be tightly regulated by intricate mechanisms to shape specific immune responses. SR-A1, as a pattern recognition receptor primarily expressed on macrophages, plays a key role in inducing inflammation for host defence against pathogens [40]. SR-A1, despite lacking intrinsic signaling motifs, can cooperate with TLR4 to recognize and phagocytose *Escherichia coli*, thereby boosting innate immune cell activation and the inflammatory response [9]. Our study unveiled a previously unrecognized mechanism by which schistosomes employ SR-A1 to enhance TLR4-driven immunoregulation in hepatic pathology, underscoring the critical role of SR-A1 in preserving immune homeostasis during schistosome infection. Of note, considering that TGF-β1 is generated as a pro-form (LAP-bound latent complex) and requires bioactivation to exert function [31], we confirmed *in vivo* and *in vitro* that the SR-A1/TLR4 axis induces the release of biologically active TGF-β1 that mediates Treg induction.

Most TLRs activate the downstream NF-κB pathway, leading to inflammatory cytokine production. Interestingly, recent evidence, including our study, demonstrates that helminths also harness TLR signaling to promote a tolerogenic phenotype in innate immune cells secreting anti-inflammatory cytokines [39]. However, the exact molecular mechanisms underlying the anti-inflammatory properties of both TLRs and SRs remain unclear. Our study identified an unknown mechanism by which two PRRs collaboratively induce an immunosuppressive phenotype of macrophages. Specifically, SR-A1 upregulates c-Jun expression to prompt its interaction with TLR4-activated p50, forming a novel transcriptional complex that significantly enhances TGF-β1 transcription in macrophages during *S. japonicum* infection. In addition, we also found that c-Jun inhibition in macrophages eliminated SR-A1-enhanced TGF-β1 expression, pointing to the key role of c-Jun in the cooperative effect of SR-A1 on TLR4-p50. However, the underlying mechanism by which SR-A1 promotes c-Jun expression requires further investigation.

Of note, our results showed that SR-A1-enhanced TGF-β1 expression in macrophages is completely reliant on p50. Moreover, inhibiting p50 in macrophages was more effective than SR-A1 deficiency in reducing TGF-β1 expression. These results suggest the essential role of TLR4-p50 in driving TGF-β1 expression. Given that p50 lacks a transcription activation domain, these results suggest that TLR4-activated p50 might cooperate with other additional unknown transcriptional co-activators to activate TGF-β1 transcription.

P50 is primarily known as a transcriptional repressor of pro-inflammatory cytokines like TNF-α and IFN-β, by competitively binding with other NF-κB subunits [41,42]. Our study, however, for the first time demonstrates that p50 also binds to the TGF-β1 promoter (a major anti-inflammatory cytokine) and promotes TGF-β1 expression with co-activator c-Jun in macrophages. Similarly, earlier studies have shown that p50 can act as a transcriptional co-activator, promoting IL-10 expression in cooperation with co-activator CBP (CREB-binding protein) [43,44]. Therefore, our study further reinforces the concept that p50 plays immunoregulatory roles via dual mechanisms, differentially regulating pro- and anti-inflammatory cytokines [42,44]. Indeed, by using p50-deficient mice, various groups have shown that p50 deficiency aggravates the immunopathology in a number of inflammatory disease models affecting the kidney, liver, lung, and brain [45–49]. Likewise, our results from *in vivo* macrophage p50 knockdown experiments reveal that p50 limits hepatic immunopathology in *S. japonicum*-infected mice. Therefore, p50 is a critical immunoregulatory transcription factor of the immune system.

Fucoidan selectively upregulated c-Jun without enhancing p50 expression in macrophages, yet required both NF-κB and AP-1 signaling to induce Tregs and ameliorate hepatic fibrosis in *S. japonicum*-infected mice. This “two-signal” requirement creates a safety checkpoint that restricts TGF-β1 induction to inflamed tissues. Importantly, from a translational perspective, our mechanistic discoveries establish fucoidan, a dietary polysaccharide isolated from marine brown seaweeds [29], as a promising immunomodulatory agent, potentially complementing standard praziquantel regimens to prevent fibrotic progression in chronic schistosomiasis. Taken together, our results demonstrate that *S. japonicum* utilizes SR-A1 to upregulate c-Jun, which cooperates with TLR4 signaling to assemble c-Jun-p50 transcription complexes binding to the TGF-β1 promoter in macrophages, revealing a discrete mechanism whereby helminths exploit the synergy between pattern recognition receptors (SR-A1 and TLR4) to enforce Treg induction and limit immunopathology in the host. These findings illuminate a hitherto unrecognized host-parasite interface wherein helminths orchestrate tolerogenic programs, offering rational targets for modulating chronic parasitic immunopathologies.

## Materials and methods

### Ethics statement and mice

The animals were handled in strict accordance with the Regulations for the Administration of Affairs Concerning Experimental Animals (1988.11.1), and all efforts were made to minimize suffering and discomfort. All experimental animal procedures were reviewed and approved by the Institutional Animal Care and Use Committee (IACUC) of Nanjing Medical University for the use of laboratory animals (Permit Number: IACUC-2110001).

Female 6- to 8-week-old wild-type C57BL/6 mice were purchased from the Model Animal Research Center of Nanjing University (Nanjing, China). *Msr1* knockout (*Msr1*^−/−^) and myeloid-specific *Msr1* knockout mice (*Msr1*^f/f^*Lyz2*^cre^) were a gift from Professor Qi Chen (Nanjing Medical University). All mice were maintained in a specific pathogen-free breeding facility.

### Cell culture and stimulations

The murine Mφ cell line RAW264.7 was obtained from the Cell Bank of the Chinese Academy of Sciences (Shanghai, China). Bone-marrow-derived macrophages (BMDMs) were generated using M-CSF (20 ng/mL; PeproTech) as described previously [50]. Cells were maintained in complete Dulbecco’s Modified Eagle’s Medium (DMEM, Gibco) supplemented with 10% (v/v) heat-inactivated fetal bovine serum (FBS, Gibco) and antibiotics (penicillin and streptomycin; Gibco). Cells transiently transfected with or without siRNAs were seeded in 12-well plates (5 × 10^**5**^ cells/mL per well) and *in vitro* stimulated with SEA (10 μg/mL), SjHSP60 (0.5 μg/mL), fucoidan (100 μg/mL), BAY 11–7082 (10 μM; Selleck), or TAK-242 (Resatorvid, 10 μM; TargetMol). After 48 hours, cells were collected for subsequent analysis.

BMDMs were stimulated with SjHSP60 (0.5 μg/mL) plus fucoidan (100 μg/mL) for 48 hours. The conditioned supernatants were collected, and mouse splenocytes were seeded into 96-well U-bottom plates at 1 × 10^6^ cells per well in a final volume of 200 μL, comprising 100 μL conditioned supernatant and 100 μL fresh complete DMEM, in the presence or absence of SB431542 (10 μM; TGF-βRI inhibitor, Selleck). After 48 hours of culture, cells were harvested, and Tregs were analyzed by flow cytometry.

### SjHSP60 expression and purification

SjHSP60 was expressed and purified by the methods previously described [18].

### Infection of mice with *S. japonicum*

Mice were infected through percutaneous exposure of the abdominal skin for 20 min to 12 *S. japonicum* cercariae (Chinese mainland strain) from infected snails (*Oncomelania hupensis*) obtained from the Jiangsu Institute of Parasitic Diseases (Wuxi, China).

### Administration of recombinant TGF-β1, anti-TGF-β1 neutralizing antibody, and fucoidan

For mice infected with *S. japonicum*, recombinant TGF-β1 (BioLegend) was administered intraperitoneally at 0.5 μg per mouse every 2 days from 6 to 8 weeks post-infection; fucoidan (Sigma-Aldrich) was administered by oral gavage at 12.5 mg per mouse every 2 days from 3 to 8 weeks post-infection; a neutralizing anti‑TGF‑β1 antibody (250 μg per mouse; BioXCell) was administered intraperitoneally every three days from 6 to 8 weeks post‑infection. All mice were euthanized 24 hours after the final injection.

### siRNA duplexes and transfection

Small interfering RNAs (siRNAs) targeting murine *Nfkb1/p105* and *c-jun* were commercially obtained from Hanbio Biotechnology. The specific siRNA sequences are as follows. *Nfkb1*/*p105* sense (5’-3’): GCAGGTATTTGACATACTAAA. *c-jun* sense (5’-3’): GGCACAGCUUAAGCAGAAATT.

Mφ (RAW264.7 cells) were seeded at 1.0 × 10^**6**^ cells/well in 6-well plates (2 mL per well). After 12 h of incubation, transfections of siRNAs were performed using CRTrans Transfection Reagent (Corues Biotechnology) according to the manufacturer's instructions. The knockdown efficiency was assayed by RT-PCR or Western Blotting at 48 hours post-transfection.

### RNA extraction and quantitative real-time PCR (RT-PCR)

Total RNA was isolated from tissues or cells with TRIzol Reagent (Invitrogen), and its concentration was measured using a NanoDrop spectrophotometer (ThermoFisher). Total isolated RNA (2 μg) from each sample was reverse-transcribed using HiScript IV All-in-One Ultra RT SuperMix (R433-01; Vazyme) following the manufacturer’s instructions. Real-time PCR was performed in a Step One Plus™ Real-Time PCR System (Applied Biosystems, CA, USA) using ChamQ SYBR Color qPCR Master Mix (Q411-02; Vazyme). Results were normalized to the housekeeping gene *Gapdh*.

The primers used are as follows: Tgfb1, forward, 5′-TGACGTCACTGGAGTTGTACGG-3′, and reverse, 5′-GGTTCATGTCATGGATGGTGC-3′; gapdh, forward, 5′-GGTGAAGGTCGGTGTGAACG-3′, and reverse, 5′-ACCATGTAGTTGAGGTCAATGAAGG-3′; Actb, forward, 5′-GGCTGTATTCCCCTCCATCG-3′, and reverse, 5′-CCAGTTGGTAACAATGCCATGT-3′.

### Western blotting

Forty-eight hours after siRNA transfection or treatment, whole-cell lysates or nuclear fractions were prepared, respectively, with cell lysis buffer (50 mM Tris-HCl [pH 8.0], 250 mM NaCl, 0.2% Nonidet P-40, 5 mM EDTA [pH 8.0], 1mM DTT, 5% glycerol) with a protease inhibitor cocktail (Roche Applied Science) or the Nuclear Extraction Kit (Millipore-Upstate, Billerica, MA) according to the manufacturer’s instructions. Protein concentrations were quantified using the DC protein assay (Bio-Rad, Hercules, CA). Equal amounts of soluble protein (30–100 μg) were fractionated by SDS-PAGE and subsequently transferred onto nitrocellulose membranes. After blocking with 5% (w/v) milk in Tris-buffered saline containing 0.1% Tween 20 (TBS-T) for 2 h, the membranes were then incubated with primary antibodies at 4°C overnight. The primary antibodies were diluted and used according to the manufacturer's instructions. After washing with TBS-T, the membranes were incubated with corresponding horseradish peroxidase-conjugated secondary antibodies, and protein bands were detected using the ChemiDoc MP imaging system (Bio-Rad). The band density was analyzed with ImageJ software (https://imagej.net/).

Antibodies used are as follows: anti-TGF-β1 (ab179695; Abcam), anti-p50 (13586; Cell Signaling Technology, CST), anti-COL1A1 (72026; CST), anti-c-Jun (24909–1-AP; Proteintech), anti-p65 (80979–1-RR; Proteintech), anti-IKKα (F0995; Selleck), anti-IKKβ (F0333; Selleck), anti-Polymerase II (NB200–598SS; NOVUS), anti-GAPDH (60004–1-lg; Proteintech), anti-SR-A1 (MCA1322; BIO-RAD), anti-H3 (F0057; Selleck), anti-IL-13 (83935–1-RR; Proteintech), anti-IL-10 (A2171; ABclonal), HRP-conjugated anti-rabbit IgG (7074; CST), and HRP-conjugated anti-mouse IgG (7076; CST).

### Active TGF-β1 measurements

Active TGF-β1 levels in mouse serum or cell-culture supernatants were measured using the LEGEND MAX Free Active TGF-β1 ELISA Kit (BioLegend) according to the manufacturer’s instructions.

### Co-immunoprecipitation

Co-immunoprecipitation (Co-IP) was performed as previously described [51]. Briefly, RAW264.7 Mφ cytosolic and nuclear proteins were extracted using NE-PER Nuclear and Cytoplasmic Extraction Reagents (Thermo Fisher Scientific) and quantified using BCA (Thermo Fisher Scientific). Cell extractions were incubated with anti-p50 (13586; CST), anti-c-Jun (24909-1-AP; Proteintech), and protein A/G plus agarose (Absin) overnight at 4 °C. The immunocomplexes were blotted with antibodies against p50 and c-Jun. Cell lysates were also immunoblotted with antibodies against p50 and c-Jun, respectively.

### CHIP analysis

Mφ was treated with 0.5 μg/mL SjHSP60 for 16 h. After cross-linking chromatin with 1% formaldehyde, cells were lysed as described above and sonicated to shear DNA. CHIP was performed using the CHIP Assay Kit (Beyotime Institute of Biotechnology, Jiangsu, China) according to the manufacturer’s protocol with anti-NF-κB p50 (Millipore-Upstate), anti-NF-κB p65 (CST), or isotype IgG (CST). Immunoprecipitated DNA was extracted using phenol-chloroform and analyzed by qPCR with primers for the promoter of TGF-β1 using FastStart SYBR Green Master Mix (Roche Applied Science). Primers for the murine *Tgfb1* promoter were 5’-ACCCTGGTGGTATACTGAG-3’ (forward) and 5’- CACGGCACTTCGGAGAGC-3’ (reverse).

### CUT&Tag-qPCR

Mφ (RAW264.7 cells) transiently transfected with or without siRNAs were stimulated with SjHSP60 (0.5 μg/mL). After 48 hours, 1 × 10^5^ cells were processed using the Hyperactive Universal CUT&Tag assay Kit (TD904; Vazyme) according to the manufacturer’s instructions. Samples were incubated overnight with primary antibodies against p50 or an IgG isotype control (1:100 dilution), followed by incubation with the corresponding secondary antibody for 1 h at room temperature. Binding of the hyperactive pA/G-transposon with the secondary antibodies was performed. Following fragmentation, DNA fragments were purified and analyzed by qPCR, with fold enrichment normalized to spike-in DNA. The following antibodies were used: anti-p50 (13586; CST), anti-p65 (80979–1-RR; Proteintech), and rabbit IgG isotype control (30000–0-AP; Proteintech). The primer sequences for the *Tgfb1* promoter were as follows: Forward: 5′-CCCTATGCCATGGACTGCAA-3′, Reverse: 5′-CCCACAGCCCTTTCTACCC-3′.

### Recombinant adeno-associated virus and *in vivo* delivery methods

An adeno-associated virus (AAV) serotype 9 vector expressing Mφ-specific *Nfkb1* shRNA (AAV-F4/80-mirR30-sh-Nfkb1), *c-jun* (AAV-F4/80-mirR30-Jun), and their corresponding empty vectors (AAV-F4/80-mirR30-sh-Ctrl and AAV-F4/80-Ctrl) were obtained as ready-to-use viral stock from Hanbio Biotechnology (Shanghai, China). The AAV9 system harbors an Mφ-specific synthetic promoter F4/80, a miR30-based shRNA targeting *Nfkb1*, and a cytomegalovirus promoter. The nucleotide sequence of the *Nfkb1* shRNA was cloned using the following sequence: 5’-GCAGGTATTTGACATACTAAA-3’. Mice were injected with 10^11^ viral particles via the tail vein three weeks after *S. japonicum* infection.

### Immunofluorescence staining and flow cytometry (FCM)

Single-cell suspensions of splenocytes or hepatic lymphocytes from mice were prepared as previously described [52]. Single cells were pretreated with anti-CD16/32 (clone 93; eBioscience) to block Fc receptors, and then stained with the indicated fluorophore-conjugated antibodies. To detect Foxp3 expression, cells were permeabilized (00-5523-00; eBioscience) and then stained with anti-Foxp3 (FJK -16s; eBioscience). After staining, samples were analyzed using a FACSVerse instrument (BD Bioscience) and FlowJo software (version 10.8.1).

Antibodies used for FCM are as follows: anti-SiglecF (S17007L; Biolegend), anti-CD11b (M1/70; BD Pharmingen), anti-F4/80 (BM8; eBioscience), anti-Ly6G (1A8; Biolegend), anti-CD45 (30-F11; eBioscience), anti-CD3 (500A2; BD Pharmingen), anti-CD4 (RM4–5; BD Pharmingen), anti-CD25 (PC61.5; eBioscience), and anti-TGF-β1 (TW7-16B4; Biolegend).

### Histopathology

Liver tissue samples from infected mice were fixed in 10% buffered formalin, followed by paraffin embedding and sectioning (4 μm).

The collagen in the liver section was stained with Masson for semi-quantitative analysis of hepatic fibrosis. Four to six random fields were acquired from each stained section under an optical microscope (Zeiss) equipped with a digital camera. The percentage of the blue-stained area and the intensity of fibrosis were assessed using ImageJ software.

### RNA-sequencing (RNA-seq) and bioinformatic analysis

Hepatic Mφ (SiglecF^−^F4/80^+^CD11b^+^) of *Msr1*^f/f^ and *Msr1*^f/f^*Lyz2*^cre^ mice were isolated by flow sorting (BD FACSAria III). Total RNA samples were extracted from Mφ and subjected to RNA-seq analysis by the BGI company (Shenzhen, China). Kyoto Encyclopedia of Genes and Genomes (KEGG) pathway enrichment analysis and heatmap construction were performed using the BGI service online.

### Statistical analysis

All statistical analyses were performed using IBM SPSS Statistics 22.0 (IBM Corp., Armonk, NY). Comparing the means of two groups was performed using Student’s *t*-test (n ≥ 5) or Welch’s *t*-test (n < 5). The differences among three or more groups were analyzed with a one-way analysis of variance (ANOVA) with an LSD post hoc test. If the *P* value is less than 0.05, the result is considered significant.

## Supporting information

S1 File Graphical Abstract. (DOCX)

S1 Fig Predominant SR-A1 expression in hepatic macrophages and its upregulation during *S. japonicum* infection. (A and B) ScRNA-seq analysis of *Msr1* expression in major cell populations of the liver from *S. japonicum*-infected mice (GSE220286). UMAP of liver cell clusters showing *Msr1* expression distribution (A); Violin plot showing *Msr1* expression across liver cell clusters (B). (C and D) *Msr1* expression in the liver of *S. japonicum*-infected patients (C) and mice (D) based on the publicly available microarray datasets (GSE61376 and GSE14367). (E) FCM analysis of SR-A1 expression on macrophages (F4/80^+^CD11b^+^Siglec-F^−^) in the liver of normal and *S. japonicum*-infected mice. Representative FCM dot plots (left) and quantification (right) are shown. Each dot represents an individual sample, and the number of dots in each group corresponds to its sample size (n). Comparing the means of two groups was performed using Welch’s *t*-test (n < 5). *P < 0.05.
(TIF)

S2 Fig The number of macrophages in *Msr1*^−/−^ and *Msr1*^f/f^*Lyz2*^cre^ mice with *S. japonicum* infection. (A and B) FCM analysis of F4/80^+^ CD11b^+^Siglec-F^−^ macrophages in the liver (A) and spleen (B) of WT, *Msr1*^−/−^, and *Msr1*^f/f^*Lyz2*^cre^ mice with or without *S. japonicum* infection. Representative FCM dot plots and quantification are shown. Each dot represents an individual sample, and the number of dots in each group corresponds to its sample size (n). The differences among groups were analyzed with ANOVA. ***P < 0.001, ns indicates not significant.
(TIF)

S3 Fig Hepatic IL-10 and IL-13 levels in *Msr1*^−/−^ and *Msr1*^f/f^*Lyz2*^cre^ mice with *S. japonicum* infection. (A and B) Hepatic IL-10 and IL-13 levels in wild-type (WT), *Msr1*^−/−^, *Msr1*^f/f^, and *Msr1*^f/f^*Lyz2*^cre^ mice with *S. japonicum* infection were quantified by Western blotting. Bar graphs show mean ± SD. Each dot represents an individual sample, and the number of dots in each group corresponds to its sample size (n). Comparing the means of two groups was performed using Student’s *t*-test (n ≥ 5). *P < 0.05, **P < 0.01, ***P < 0.001, ns indicates not significant.
(TIF)

S4 Fig AlphaFold-based structural modeling of the SjHSP60-SR-A1 interaction. **(A)** Predicted interaction model between SjHSP60 and SR-A1. (B) Confidence scores of the predicted complex. The most probable binding model shows pLDDT = 58.2, pTM = 0.488, and ipTM = 0.172.
(TIF)

S5 Fig TLR4 and SR-A1 are required for SjHSP60- and Fucoidan-induced macrophage TGF-β1 expression, respectively. (A) BMDMs from wild-type mice were stimulated with SjHSP60 (0.5 μg/mL) and/or TAK-242 (10 μM; TLR4 inhibitor), or left untreated, for 48 hours. (B) BMDMs from *Msr1*^f/f^*Lyz2*^cre^ mice were stimulated with or without fucoidan (100 μg/mL) for 48 hours. *Tgfb1* expression in macrophages was assayed by RT-PCR. Bar graphs show mean ± SD. Each dot represents an individual sample, and the number of dots in each group corresponds to its sample size (n). The differences among groups were analyzed with ANOVA. *P < 0.05, **P < 0.01, ***P < 0.001.
(TIF)

S6 Fig SjHSP60/Fucoidan-stimulated macrophages promote Treg generation via TGF-β1. (A) BMDMs from wild-type C57BL/6 mice were stimulated with SjHSP60 (0.5 μg/mL) and fucoidan (100 μg/mL) for 48 hours, and active TGF-β1 in the cell culture supernatant was quantified by ELISA. (B) BMDMs stimulated as in (A) were co-cultured with splenocytes that had been pretreated with or without SB431542 (10 μM; TGF-βRI inhibitor) for 48 hours, and CD4^+^CD25^+^Foxp3^+^ Tregs were then analyzed by FCM. Bar graphs show mean ± SD. Each dot represents an individual sample, and the number of dots in each group corresponds to its sample size (n). Comparing the means of two groups was performed using Student’s *t*-test (n ≥ 5). The differences among three or more groups were analyzed with ANOVA. *P < 0.05, ***P < 0.001.
(TIF)

S7 Fig IKKα and IKKβ expression in macrophages following SjHSP60 stimulation. (A and B) RAW264.7 macrophages were cultured with or without SjHSP60 (0.5 μg/mL) for 48 hours. IKKα and IKKβ expression in macrophages was determined by Western blotting. Each dot represents an individual sample, and the number of dots in each group corresponds to its sample size (n). Comparing the means of two groups was performed using Welch’s *t*-test (n < 5). **P < 0.01, ns indicates not significant.
(TIF)

S8 Fig Elevated c-Jun expression in macrophages from *S. japonicum*-infected mice. (A and B) FCM analysis of c-Jun expression in macrophages (F4/80^+^CD11b^+^Siglec-F^−^) from the liver (A) and spleen (B) of normal and *S. japonicum*-infected mice. Representative FCM histograms and quantification are shown. Bar graphs show mean ± SD. Each dot represents an individual sample, and the number of dots in each group corresponds to its sample size (n). Comparing the means of two groups was performed using Welch’s *t*-test (n < 5). *P < 0.05, **P < 0.01.
(TIF)

S9 Fig Schematic illustration of the experimental design. *S. japonicum*-infected mice were orally administered fucoidan (500 mg/kg body weight) and/or intraperitoneally injected with BAY 11–7082 (3 mg/kg) and T5224 (3 mg/kg), as indicated.
(TIF)

S1 Raw Blot Image The raw data of western blot in this study. (PDF)

S1 Raw Data The raw data supporting each of the manuscript Figures are contained in this Excel file. (XLSX)
