# Supplementary material for: SR-A1 cooperates with TLR4-NF-κB signaling to drive macrophage TGF-β1 expression and Treg induction during schistosome infection
Source: PLoS Pathog. 2026 Oct 30;22(10):e1014732. doi: 10.1371/journal.ppat.1014732 (PMC13750658; doi:10.1371/journal.ppat.1014732)
Supplement: Supplementary file 1 [file ppat.1014732.s011.pdf]

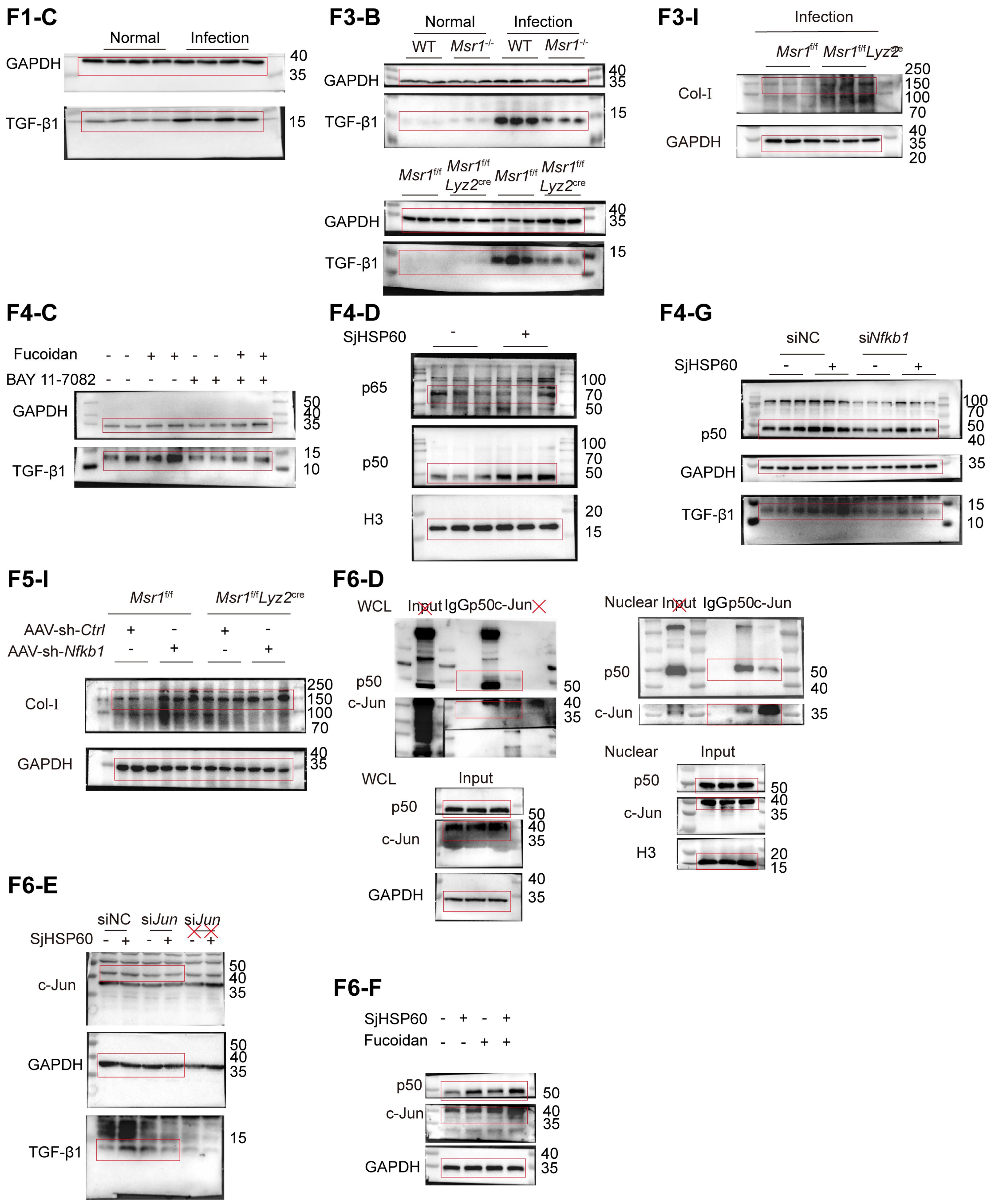
F1-C
Normal
Infection
GAPDH
TGF-β1
40
35
15
F3-B
Normal
Infection
WT
Msr1-/-
GAPDH
TGF-β1
Msr1f/f
Msr1f/f Lyz2cre
F3-I
Infection
Col-I
GAPDH
250
150
100
70
F4-C
Fucoidan
BAY 11-7082
F4-D
SjHSP60
p65
p50
H3
F4-G
siNC
siNfkb1
F5-I
AAV-sh-Ctrl
AAV-sh-Nfkb1
F6-D
WCL
Input
IgG
p50
c-Jun
Nuclear
F6-E
siJun
F6-F

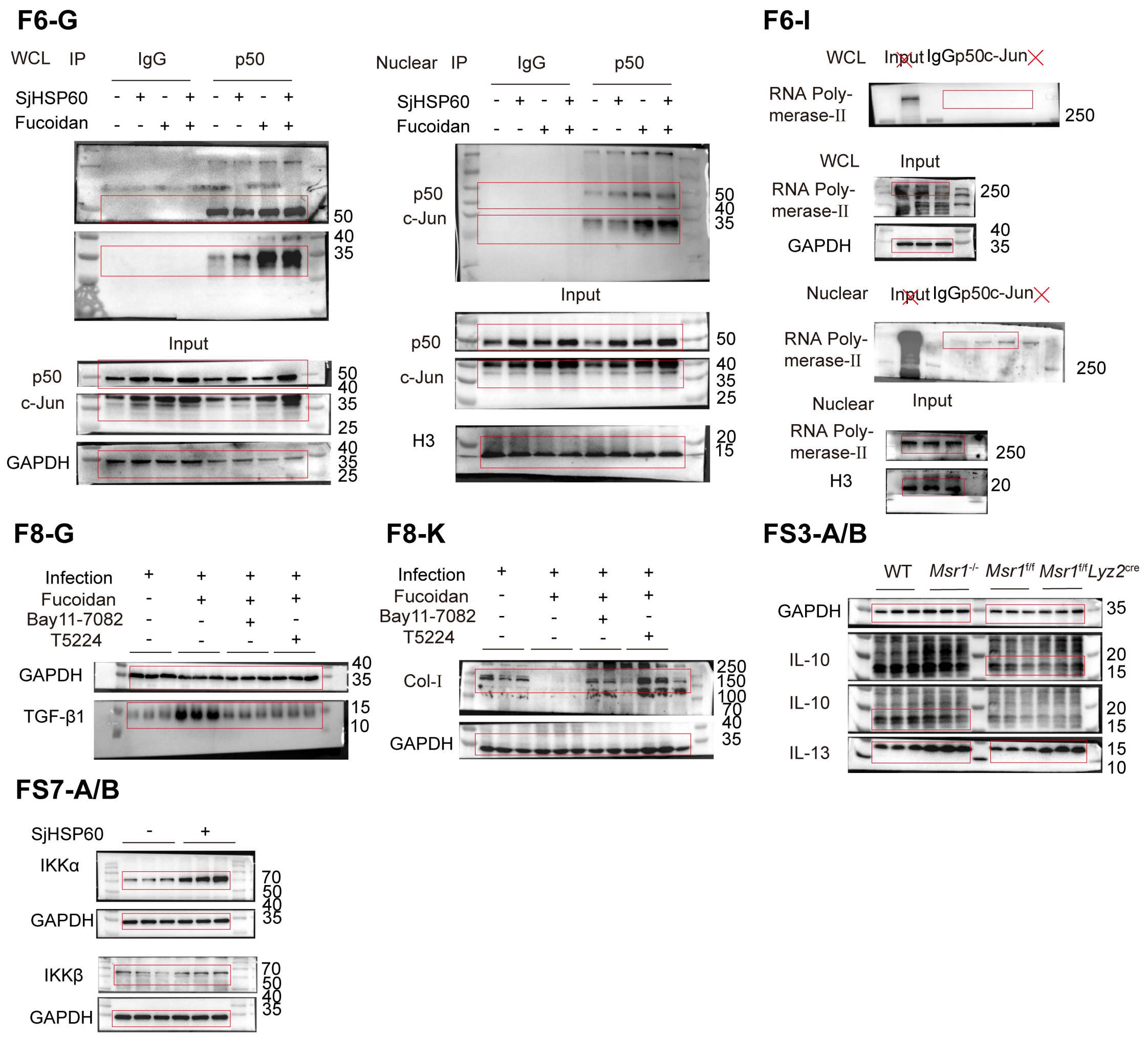
F6-G
WCL IP IgG p50
SjHSP60
Fucoidan
Input
p50
c-Jun
GAPDH
Nuclear IP IgG p50
H3
F6-I
WCL Input IgG p50 c-Jun
RNA Poly-merase-II
Nuclear Input IgG p50 c-Jun
F8-G
Infection
Fucoidan
Bay11-7082
T5224
TGF-β1
F8-K
Col-I
FS3-A/B
WT
Msr1-/-
Msr1f/f
Msr1f/fLyz2cre
IL-10
IL-13
FS7-A/B
IKKα
IKKβ
