# Supplementary material for: SR-A1 cooperates with TLR4-NF-κB signaling to drive macrophage TGF-β1 expression and Treg induction during schistosome infection
Source: PLoS Pathog. 2026 Oct 30;22(10):e1014732. doi: 10.1371/journal.ppat.1014732 (PMC13750658; doi:10.1371/journal.ppat.1014732)
Supplement: Supplementary file 2 [file ppat.1014732.s014.pdf]

We truly appreciate the valuable opportunity to revise our manuscript. We carefully considered the constructive comments provided by the reviewers and the editors.

We have performed additional experiments, added new figures (Figure 1D, Figures 1G-1H, Figure 3C, Figure 4F, Figure 4I, Figures S3A-S3B, and Figures S6A-S6B), and extensively revised our manuscript. All the newly acquired results further support the conclusions described in our manuscript. We hope that the editors and reviewers find our responses and revisions satisfactory and that the revised manuscript fulfills *PLOS Pathogen*s' requirements for publication.

Our point-by-point responses to all the comments are described below.

(NOTE*: All the page and line numbers mentioned below refer to the manuscript version with track changes.)*

Reviewers' Comments:

Reviewer's Responses to Questions

Part I – Summary

Please use this section to discuss strengths/weaknesses of study, novelty/significance, general execution and scholarship.

Reviewer #1: In this manuscript, the authors show investigate a macrophage pathway by which S. japonicum induces a TGF-β-dependent immunosuppressive pathway. They show that this pathway controls regulatory T cell and fibrotic responses in infection. They investigate the signalling pathway that mediates this effect and find that macrophage SR-A1 and TLR4 activation leads to the activation of NFKB p50 and cJun activation and complex formation, resulting in transcriptional upregulation of TGF-β. Finally, they show that fucoidan can also stimulate SR-A1 to induce regulatory responses.

This study has strengths in the range of techniques used and robustness of approaches. The focus on determining the molecular pathway of action is commendable and allows strong conclusions to be made.

Response: We sincerely thank Reviewer #1 for the thorough evaluation and positive comments on our work.

Reviewer #2: This manuscript investigates whether SR-A1 plays a regulatory role in TLR4-driven TGF-β1 expression in macrophages during Schistosoma japonicum infection, and explores the underlying molecular mechanisms. The authors propose a model in which SR-A1 promotes c-Jun expression, enabling interaction with TLR4-induced NF-κB p50, thereby facilitating formation of a c-Jun–p50 transcriptional complex at the Tgfb1 promoter. This axis enhances TGF-β1 production, promotes Treg induction, and mitigates liver immunopathology. Overall, the study is well-executed and conceptually interesting, presenting a dual innate receptor cooperation model (SR-A1 + TLR4)

that shifts macrophages toward a tolerogenic phenotype. The work is relevant to the field of helminth immunoregulation and is supported by solid experimental breadth and a coherent mechanistic framework. However, several major issues need to be addressed, particularly regarding mechanistic completeness, statistical rigor, and integration with established pathways of schistosomiasis immunopathology.

Response: We sincerely thank Reviewer #2 for the thorough evaluation and for recognizing the conceptual interest and experimental breadth of our study. We have carefully addressed all raised concerns regarding mechanistic completeness, statistical rigor, and integration with established immunopathological pathways, with detailed responses provided below.

Part II – Major Issues: Key Experiments Required for Acceptance

Please use this section to detail the key new experiments or modifications of existing experiments that should be **absolutely** required to validate study conclusions.

Generally, there should be no more than 3 such required experiments or major modifications for a "Major Revision" recommendation. If more than 3 experiments are necessary to validate the study conclusions, then you are encouraged to recommend "Reject".

Reviewer #1: TGF-β is regulated mainly at the post-transcriptional level, due to its production in a pro-form, followed by cleavage and release from a latency associated peptide. This should be discussed in the text. Furthermore, experimental data should be added to measure that active TGF-beta protein is released from the stimulated macrophages, using e.g. a bioassay of supernatants, or transfer of macrophage supernatants to T cells to show that this can induce regulatory T cell responses in a TGF-B receptor signalling-dependent manner.

Response: We appreciate the reviewer for this valuable suggestion regarding the post-transcriptional regulation of TGF-β1. To address this concern, we have performed additional experiments to directly demonstrate that stimulated macrophages release active TGF-β1 capable of inducing Tregs (new Figure S6). Specifically, we found that the supernatant derived from BMDMs co-stimulated with SjHSP60 and fucoidan exhibited significantly elevated levels of active TGF-β1 (new Figure S6A). Furthermore, using a TGF-βRI inhibitor, we demonstrated that this macrophage-conditioned supernatant markedly promoted Treg generation in splenocytes in a TGF-β1 signaling-dependent manner (new Figure S6B). Importantly, we additionally observed that *S. japonicum* infection elevated serum active TGF-β1 levels (new Figure 1D), which were significantly attenuated upon macrophage-specific deletion of SR-A1 (new Figure 3C).

These results confirm that the SR-A1/TLR4 axis drives the release of biologically active TGF-β1, thereby functionally enabling Treg induction. We have added these newly acquired data to new Figures (S6, 1D, and 3C) and revised the manuscript (page 7 lines 10-11, page 8 lines 17-19, and page 10 lines 25-28). In addition, we have also added some relevant discussion regarding this concern in our revised manuscript (page 17 lines 24-27) **as follows:** "*Of note, considering that TGF-β1 is generated as a pro-form (LAP-bound latent complex) and requires bioactivation to exert*

function [31], we confirmed in vivo and in vitro that the SR-A1/TLR4 axis induces the release of biologically active TGF-β1 that mediates Treg induction."

Related to the above, anti-TGF-β is applied in vivo but only data on fibrosis is shown (Fig1G) - regulatory T cell responses should also be shown.

Response: We fully agree with the reviewer. To address this, we have performed additional experiments showing that *in vivo* TGF-β1 neutralization using anti-TGF-β1 significantly reduced both the proportion and absolute number of Tregs in the liver and spleen of *S. japonicum*-infected mice (new Figures 1G and 1H).

We have added these newly acquired data to new Figures 1G and 1H and revised the manuscript (page 7 lines 13-14). These findings are consistent with the well-established role of TGF-β1 as a critical driver of Treg differentiation and maintenance, and they directly complement the fibrosis data.

Reviewer #2: Major comments:

1. A major conceptual gap is the lack of integration with IL-13–driven fibrosis, which is widely recognized as the dominant pathway in schistosomiasis immunopathogenesis. The manuscript focuses on TGF-β1–mediated regulation but does not address: How TGF-β1 induced via SR-A1/TLR4 crosstalk interacts with IL-13 signaling. Whether Treg induction is associated with changes in IL-10 and/or suppression of IL-13 responses. This is particularly important because Liver fibrosis in schistosomiasis is primarily driven by Type 2 cytokines (especially IL-13). For a better understanding of the mechanisms, the authors should provide data or discussion linking their findings to IL-13–mediated pathology, and clarify whether the observed protection is due to modulation of Type 2 responses.

Response: We thank the reviewer for this important comment. To address this, we conducted additional Western blot analyses and found that IL-10 remained unchanged, whereas IL-13 was significantly increased in the livers of *Msr1*$^{-/-}$ and *Msr1*$^{f/f}$*Lyz2*^{cre} mice infected with *S. japonicum* (new Figure S3A and S3B). These findings suggest that SR-A1/TLR4-driven Treg-mediated protection is dissociated from IL-10 and involves suppression of Type 2 immune responses (IL-13, etc.).

To further confirm the specific mechanism mediated by TGF-β1, we applied a TGF-RI inhibitor in an *in vitro* Treg-inducing co-culture system, in which splenocytes were cultured with the conditioned supernatant from BMDMs co-stimulated with SjHSP60 and fucoidan, demonstrating that this induction specially relies on TGF-β1 signaling (new Figure S6B).

Collectively, we propose that the SR-A1/TLR4 axis induces TGF-β1, which expands Tregs to constrain IL-13-producing effector cells, thereby limiting IL-13-driven fibrosis. In the absence of SR-A1, impaired TGF-β1-Treg signaling leads to unchecked IL-13 production and exacerbated liver fibrosis.

We have added these new data as new Figures S3 and S6B, and revised the manuscript (page 9 lines 1-7 and page 10 lines 25-28). In addition, we have also added some relevant discussion regarding this concern in our revised manuscript (page 16 lines 24-30) **as follows:** *"IL-13, as a*

pivotal type 2 immune effector molecule, is the dominant pro-fibrogenic cytokine for driving hepatic fibrosis in schistosomiasis [35, 36]. As expected, we observed higher hepatic IL-13 levels in Msr1$^{-/-}$ and Msr1$^{f/f}$Lyz2^{cre} mice infected with S. japonicum. Therefore, our findings provide mechanistic evidence that SR-A1-driven TGF-β1 production, which expands Tregs and operates hepatic protection, at least in part, by constraining type 2 immune responses and limiting IL-13-driven fibrogenesis."

2. The statistical approach requires substantial improvement and clarification. In several experiments (e.g., Figure 1), comparisons are made between two groups with small sample sizes (n = 3–5 mice) with significant deviation across the datasets. Despite this Parametric t-tests are used without justification. Differences appear modest, raising concerns about robustness. Justify the use of parametric tests or use non-parametric tests (e.g., Mann-Whitney) where appropriate.

Response: Thank the reviewer for this important and constructive comment. We fully agree that statistical rigor is essential, particularly when sample sizes are modest. We have carefully re-evaluated all statistical analyses and revised them as follows:

1. Experiments with increased sample size (n ≥ 5 per group): Student's *t*-test

For experiments where we were able to increase the sample size to n ≥ 5 per group by pooling samples across independent replicate experiments, we retained the use of Student's *t*-test.

2. Experiments where sample size remains n < 5 per group: we have replaced the standard *t*-test with Welch's *t*-test (unequal variances t-test). Welch's *t*-test was chosen because: (i) It does not assume equal variances between groups, making it more robust when sample sizes are small and variance heterogeneity is present; (ii) It provides a more conservative and reliable estimate of significance under these conditions compared to the standard Student's t-test.

Why Welch's t-test over Mann-Whitney U: We appreciate the suggestion but opted against Mann-Whitney U for our data for two reasons. (i) When n = 3~4 per group, the Mann-Whitney U test is based on ranks rather than raw values. Since the data already provide limited information at such small sample sizes, rank transformation further reduces statistical efficiency, making it difficult to achieve statistical significance and thereby increasing the risk of Type II error; (ii) our primary endpoints are continuous, ratio-scale variables, and our scientific question concerns differences in mean expression levels between groups. Welch's *t*-test directly tests differences in means, which is consistent with this question, and it maintains good control of Type I error even when variances are unequal. In contrast, Mann-Whitney U is rank-based and tests for distributional location or stochastic superiority, and its results do not necessarily correspond to differences in means.

The Methods section and figure legends have been updated accordingly. We believe these revisions strengthen the statistical robustness of our findings and hope they satisfactorily address the concerns.

3. In Figure 2, the authors identify a population of TGF-β1^{+} SiglecF^{+} F4/80^{+} cells. SiglecF is a canonical eosinophil marker. These cells may represent eosinophils rather than macrophages, or a mixed/ambiguous population?

Response: We sincerely appreciate the reviewer's insightful comment. Literature evidence indicates that F4/80 and SiglecF can be reciprocally expressed by macrophages and eosinophils under certain conditions (PMID: 1721083, 27683748, and 23023392), leading to ambiguous gating. To address this concern rigorously, we have revised our gating strategy as follows.

Given that granulocytes (including eosinophils and neutrophils) are characterized by their abundant cytoplasmic granules, resulting in high SSC signals, we first utilized SSC to segregate granulocytes (high SSC) from agranular immune cells (low-to-intermediate SSC, such as macrophages and lymphocytes). This allows us to establish distinct populations based on intrinsic cellular granularity before applying lineage-specific markers.

Importantly, this improved analysis did not alter our main findings; we still conclude that macrophages contribute predominantly to increased TGF-β1 expression in the liver of *S. japonicum*-infected mice (Figure 2E). We are grateful to the reviewer for raising this important methodological consideration, which has enabled us to significantly improve the accuracy and rigor of our analysis.

Part III – Minor Issues: Editorial and Data Presentation Modifications

Please use this section for editorial suggestions as well as relatively minor modifications of existing data that would enhance clarity.

Reviewer #1: The description of animal experiments and presentation of data should be improved. All graphs depicting animal experiments should show individual replicates, rather than mean+/-SD only. Likewise, numbers of replicates should be described in the figure legend, rather than stating e.g. "3~5 replicates per group".

Response: We sincerely thank the reviewer for this constructive suggestion. We have revised all graphs to display individual data points alongside the mean ± SD, with the number of dots in each group corresponding to the sample size (n).

TGF-beta also acts on fibroblasts and is pro-fibrotic, therefore it is surprising that increased TGF-β production leads to reduced fibrosis - this dichotomy should be discussed.

Response: We sincerely appreciate the reviewer's insightful comment regarding the apparent dichotomy that warrants careful discussion.

While TGF-β1 is canonically recognized as a potent pro-fibrotic cytokine, its biological effects are highly context-dependent (PMID: 38926762). In schistosomiasis, studies have demonstrated that the TGF-β1/Smad signaling pathway plays a comparatively minor role in driving fibrosis, whereas IL-13 emerges as the predominant pro-fibrogenic cytokine (PMID: 22593760). Crucially, our *in vivo* TGF-β1 neutralization experiments provided direct functional evidence for Treg induction and the anti-fibrotic role of TGF-β1 in a schistosomiasis model. These results suggest that, in the context of *S. japonicum* infection, macrophage-derived TGF-β1 functions primarily as an immunoregulatory cytokine that promotes Treg differentiation to dampen excessive granulomatous inflammation, thereby indirectly limiting fibrosis progression. Thus, the apparent paradox is reconciled by the dominant immunosuppressive function of TGF-β1 overriding its canonical pro-fibrotic effect in this particular pathological setting.

We have expanded the Discussion to address this dichotomy **as follows** (page 16 lines 16-24): *"Although TGF-β1 is widely recognized as a canonical pro-fibrotic cytokine, its function is highly context-dependent [34]. In S. japonicum infection, our results demonstrated that host liver macrophages are driven to robustly express TGF-β1 and become the main source of TGF-β1 in the pathogenic liver microenvironment, with this cytokine primarily acting as an immunoregulator by inducing Tregs to dampen excessive granulomatous inflammation, thereby indirectly alleviating fibrosis."*

Between figures 2 and 3, the study moves from studying TGF-β production to focusing specifically on SR-A1. This focus was not sufficiently explained - why pick this pathway? There is no doubt that this pathway is important, however this was not logically or clearly explained in the text why this pathway, rather than any other, was selected.

Response: We thank the reviewer for this comment. While the rationale for selecting SR-A1 is presented in the Introduction section, we agree that a brief logical transition was missing in the Results section. We have now added a short bridging paragraph to clarify why we focused on SR-A1 as a potential regulator of macrophage TGF-β1 expression **as follows** (page 8 lines 3-6): "*Given that SR-A1 is predominantly expressed on tissue macrophages and upregulated in anti-inflammatory macrophages characterized by high expression of immunosuppressive cytokines including TGF-β1, we hypothesized that SR-A1 regulates TGF-β1 production in hepatic macrophages during S. japonicum infection.*"

Reviewer #2: Minor comments:

4. The authors should improve clarity in figure legends, especially regarding: Statistical tests used and Sample sizes.

Response: We thank the reviewer for this suggestion. We have revised all figure legends to explicitly state the statistical tests used and the exact sample sizes (n) for each group or condition.
