# Supplementary material for: SR-A1 cooperates with TLR4-NF-κB signaling to drive macrophage TGF-β1 expression and Treg induction during schistosome infection
Source: PLoS Pathog. 2026 Oct 30;22(10):e1014732. doi: 10.1371/journal.ppat.1014732 (PMC13750658; doi:10.1371/journal.ppat.1014732)
Supplement: Peer review history

# Peer review: Decision Letter 0

**DOI:** 10.1371/journal.ppat.1014732.r001

**Authors:** Edward Mitre, Álvaro Acosta-Serrano

21 May 2026

SR-A1 cooperates with TLR4-NF-κB signaling to drive macrophage TGF-β1 expression and Treg induction during schistosome infection

PLOS Pathogens

Dear Dr. Su,

Thank you for submitting your manuscript to PLOS Pathogens. After careful consideration, we feel that it has merit but does not fully meet PLOS Pathogens's publication criteria as it currently stands. Therefore, we invite you to submit a revised version of the manuscript that addresses the points raised during the review process.

Please submit your revised manuscript by Jul 20 2026 11:59PM. If you will need more time than this to complete your revisions, please reply to this message or contact the journal office at plospathogens@plos.org. When you're ready to submit your revision, log on to https://www.editorialmanager.com/ppathogens/ and select the 'Submissions Needing Revision' folder to locate your manuscript file.

Please include the following items when submitting your revised manuscript:

* A letter that responds to each point raised by the editor and reviewer(s). You should upload this letter as a separate file labeled 'Response to Reviewers'. This file does not need to include responses to any formatting updates and technical items listed in the 'Journal Requirements' section below.

* A marked-up copy of your manuscript that highlights changes made to the original version. You should upload this as a separate file labeled 'Revised Manuscript with Track Changes'.

* An unmarked version of your revised paper without tracked changes. You should upload this as a separate file labeled 'Manuscript'.

If you would like to make changes to your financial disclosure, competing interests statement, or data availability statement, please make these updates within the submission form at the time of resubmission. Guidelines for resubmitting your figure files are available below the reviewer comments at the end of this letter.

We look forward to receiving your revised manuscript.

Kind regards,

Álvaro Acosta-Serrano

Academic Editor

PLOS Pathogens

Edward Mitre

Section Editor

Editor-in-Chief

PLOS Pathogens

orcid.org/0000-0003-2946-9497

Editor-in-Chief

PLOS Pathogens

orcid.org/0000-0002-7699-2064

**Journal Requirements:**

If the reviewer comments include a recommendation to cite specific previously published works, please review and evaluate these publications to determine whether they are relevant and should be cited. There is no requirement to cite these works unless the editor has indicated otherwise.

1) Please ensure that the CRediT author contributions listed for every co-author are completed accurately and in full.

At this stage, the following Authors/Authors require contributions: Junyao Shen, Chuanhong Huang, Ziling Wang, Weijie Xue, Yu Wu, Tianyu Fan, Chenxu Mao, Jiaojiao Yu, Yalin Li, Lei Xu, Jifeng Zhu, Xiaojun Chen, Yang Dai, Zhigang Lei, Sha Zhou, and Chuan Su. Please ensure that the full contributions of each author are acknowledged in the "Add/Edit/Remove Authors" section of our submission form.

The list of CRediT author contributions may be found here: https://journals.plos.org/plospathogens/s/authorship#loc-author-contributions

2) Please provide an Author Summary. This should appear in your manuscript between the Abstract (if applicable) and the Introduction, and should be 150-200 words long. The aim should be to make your findings accessible to a wide audience that includes both scientists and non-scientists. Sample summaries can be found on our website under Submission Guidelines:

https://journals.plos.org/plospathogens/s/submission-guidelines#loc-parts-of-a-submission

3) We do not publish any copyright or trademark symbols that usually accompany proprietary names, eg ©,  ®, or TM  (e.g. next to drug or reagent names). Therefore please remove all instances of trademark/copyright symbols throughout the text, including:

- TM on page: 26.

4) Please amend your detailed Financial Disclosure statement. This is published with the article. It must therefore be completed in full sentences and contain the exact wording you wish to be published.

1) State the initials, alongside each funding source, of each author to receive each grant. For example: "This work was supported by the National Institutes of Health (####### to AM; ###### to CJ) and the National Science Foundation (###### to AM)."

2) State what role the funders took in the study. If the funders had no role in your study, please state: "The funders had no role in study design, data collection and analysis, decision to publish, or preparation of the manuscript."

3) If any authors received a salary from any of your funders, please state which authors and which funders..

If you did not receive any funding for this study, please simply state: u201cThe authors received no specific funding for this work.u201d

5) We have amended your Competing Interest statement to comply with journal style. We kindly ask that you double check the statement and let us know if anything is incorrect.

6) PLOS now requires that authors provide the original uncropped and unadjusted images underlying all blot or gel results reported in a submission’s figures or Supporting Information files. This policy and the journal’s other requirements for blot/gel reporting and figure preparation are described in detail at https://journals.plos.org/plosone/s/figures#loc-blot-and-gel-reporting-requirements and https://journals.plos.org/plosone/s/figures#loc-preparing-figures-from-image-files.

When you submit your revised manuscript, please ensure that your figures adhere fully to these guidelines and provide the original underlying images for all blot or gel data reported in your submission. See the following link for instructions on providing the original image data: https://journals.plos.org/plosone/s/figures#loc-original-images-for-blots-and-gels.

In your cover letter, please note whether your blot/gel image data are in Supporting Information or posted at a public data repository, provide the repository URL if relevant, and provide specific details as to which raw blot/gel images, if any, are not available. Email us at customercare@plos.org if you have any questions.

**Reviewers' Comments:**

Reviewer's Responses to Questions

**Part I - Summary**

Please use this section to discuss strengths/weaknesses of study, novelty/significance, general execution and scholarship.

Reviewer #1: In this manuscript, the authors show investigate a macrophage pathway by which S. japonicum induces a TGF-b-dependent immunosuppressive pathway. They show that this pathway controls regulatory T cell and fibrotic responses in infection. They investigate the signalling pathway that mediates this effect and find that macrophage SR-A1 and TLR4 activation leads to the activation of NFKB p50 and cJun activation and complex formation, resulting in transcriptional upregulation of TGFB. Finally, they show that fucoidan can also stimulate SR-A1 to induce regulatory responses.

This study has strengths in the range of techniques used and robustness of approaches. The focus on determining the molecular pathway of action is commendable and allows strong conclusions to be made.

Reviewer #2: This manuscript investigates whether SR-A1 plays a regulatory role in TLR4-driven TGF-β1 expression in macrophages during Schistosoma japonicum infection, and explores the underlying molecular mechanisms. The authors propose a model in which SR-A1 promotes c-Jun expression, enabling interaction with TLR4-induced NF-κB p50, thereby facilitating formation of a c-Jun–p50 transcriptional complex at the Tgfb1 promoter. This axis enhances TGF-β1 production, promotes Treg induction, and mitigates liver immunopathology. Overall, the study is well-executed and conceptually interesting, presenting a dual innate receptor cooperation model (SR-A1 + TLR4) that shifts macrophages toward a tolerogenic phenotype. The work is relevant to the field of helminth immunoregulation and is supported by solid experimental breadth and a coherent mechanistic framework. However, several major issues need to be addressed, particularly regarding mechanistic completeness, statistical rigor, and integration with established pathways of schistosomiasis immunopathology.

**********

**Part II – Major Issues: Key Experiments Required for Acceptance**

Please use this section to detail the key new experiments or modifications of existing experiments that should be absolutely required to validate study conclusions.

Generally, there should be no more than 3 such required experiments or major modifications for a "Major Revision" recommendation. If more than 3 experiments are necessary to validate the study conclusions, then you are encouraged to recommend "Reject".

Reviewer #1: TGF-B is regulated mainly at the post-transcriptional level, due to its production in a pro-form, followed by cleavage and release from a latency associated peptide. This should be discussed in the text. Furthermore, experimental data should be added to measure that active TGF-beta protein is released from the stimulated macrophages, using e.g. a bioassay of supernatants, or transfer of macrophage supernatants to T cells to show that this can induce regulatory T cell responses in a TGF-B receptor signalling-dependent manner.

Related to the above, anti-TGFB is applied in vivo but only data on fibrosis is shown (Fig1G) - regulatory T cell responses should also be shown.

Reviewer #2: Major comments:

1. A major conceptual gap is the lack of integration with IL-13–driven fibrosis, which is widely recognized as the dominant pathway in schistosomiasis immunopathogenesis. The manuscript focuses on TGF-β1–mediated regulation but does not address: How TGF-β1 induced via SR-A1/TLR4 crosstalk interacts with IL-13 signaling Whether Treg induction is associated with changes in IL-10 and/or suppression of IL-13 responses This is particularly important because Liver fibrosis in schistosomiasis is primarily driven by Type 2 cytokines (especially IL-13). For a better understanding of the mechanisms, the authors should provide data or discussion linking their findings to IL-13–mediated pathology, and clarify whether the observed protection is due to modulation of Type 2 responses

2. The statistical approach requires substantial improvement and clarification. In several experiments (e.g., Figure 1), comparisons are made between two groups with small sample sizes (n = 3–5 mice) with significant deviation across the datasets. Despite this Parametric t-tests are used without justification. Differences appear modest, raising concerns about robustness. Justify the use of parametric tests or use non-parametric tests (e.g., Mann-Whitney) where appropriate

3. In Figure 2, the authors identify a population of TGF-β1⁺ SiglecF⁺ F4/80⁺ cells. SiglecF is a canonical eosinophil marker. These cells may represent eosinophils rather than macrophages, or a mixed/ambiguous population?

**********

**Part III – Minor Issues: Editorial and Data Presentation Modifications**

Please use this section for editorial suggestions as well as relatively minor modifications of existing data that would enhance clarity.

Reviewer #1: The description of animal experiments and presentation of data should be improved. All graphs depicting animal experiments should show individual replicates, rather than mean+/-SD only. Likewise, numbers of replicates should be described in the figure legend, rather than stating e.g. "3~5 replicates per group".

TGF-beta also acts on fibroblasts and is pro-fibrotic, therefore it is surprising that increased TGFB production leads to reduced fibrosis - this dichotomy should be discussed.

Between figures 2 and 3, the study moves from studying TGF-B production to focussing specifically on SR-A1. This focus was not sufficiently explained - why pick this pathway? There is no doubt that this pathway is important, however this was not logically or clearly explained in the text why this pathway, rather than any other, was selected.

Reviewer #2: Minor comments:

4. The authors should improve clarity in figure legends, especially regarding: Statistical tests used and Sample sizes

**********

PLOS authors have the option to publish the peer review history of their article (what does this mean?). If published, this will include your full peer review and any attached files.

If you choose “no”, your identity will remain anonymous but your review may still be made public.

**Do you want your identity to be public for this peer review?**  For information about this choice, including consent withdrawal, please see our Privacy Policy.

Reviewer #1: **Yes:**  Henry McSorley

Reviewer #2: No

[NOTE: If reviewer comments were submitted as an attachment file, they will be attached to this email and accessible via the submission site. Please log into your account, locate the manuscript record, and check for the action link "View Attachments". If this link does not appear, there are no attachment files.]

**Figure resubmission:**

**Reproducibility:**

?>

# Author comment: Author response to Decision Letter 1

**DOI:** 10.1371/journal.ppat.1014732.r002

21 Sep 2026

Attachment Submitted filename: Response to Reviewers_PPATHOGENS-D-26-00750.pdf

# Peer review: Decision Letter 1

**DOI:** 10.1371/journal.ppat.1014732.r003

**Authors:** Edward Mitre, Álvaro Acosta-Serrano

16 Oct 2026

Dear Prof. Su,

We are pleased to inform that your manuscript, "SR-A1 cooperates with TLR4-NF-κB signaling to drive macrophage TGF-β1 expression and Treg induction during schistosome infection", has been editorially accepted for publication at PLOS Pathogens.

Before your manuscript can be formally accepted and sent to production, you will need to complete our formatting changes, which you will receive by email within a week. Please note that your manuscript will not be scheduled for publication until you have made the required changes.

IMPORTANT NOTES

(1) Copyediting and Proofreading: The corresponding author will receive a typeset proof for review, to ensure errors have not been introduced during production. Please review the PDF proof of your manuscript carefully, as this is the last chance to correct any errors. Please note that major changes, or those which affect the scientific understanding of the work, will likely cause delays to the publication date of your manuscript.

(2) Appropriate Figure Files: Please remove all name and figure # text from your figure files. Please also take this time to check that your figures are of high resolution, which will improve the readbility of your figures and help expedite your manuscript's publication. Please note that figures must have been originally created at 300dpi or higher. Do not manually increase the resolution of your files. For instructions on how to properly obtain high quality images, please review our Figure Guidelines, with examples at: http://journals.plos.org/plospathogens/s/figures.

We encourage you to use PACE (http://pace.apexcovantage.com/), a figure-checking tool which helps authors ensure all figures meet PLOS requirements so that figures can publish in the highest quality. PACE is a digital diagnostic and conversion tool for figure files. It will provide information about any failed check(s) and, if able, will automatically convert the figure into an acceptable file that passes quality checks.

To use PACE, you must first register as a user. Then, login and navigate to the UPLOAD tab, where you will find detailed instructions on how to use the tool.

(3) Striking Image: Please upload a striking still image to accompany your article if one is available (you can include a new image or an existing one from within your manuscript). Should your paper be accepted, this image will be considered for our monthly issue image and may also appear on our website to feature your article. Please upload this as a separate file, selecting "striking image" as the file type upon upload. Please also include a separate "Other" file with a caption, including credits and any potential copyright information. Please do not include the caption in the main article file. If your image is from someone other than yourself, please ensure that the artist has read and agreed to the terms and conditions of the Creative Commons Attribution License at http://journals.plos.org/plospathogens/s/content-license. Please note that PLOS cannot publish copyrighted images.

(4) Press Release or Related Media: If you or your institution will be preparing press materials for this manuscript, please inform our press team in advance at plospathogens@plos.org as soon as possible. We ask that you contact us within one week to plan ahead of our fast Production schedule. If you need to know your paper's publication date for related media purposes, you must coordinate with our press team, and your manuscript will remain under a strict press embargo until the publication date and time.

(5) Update your Profile Information: Now that your manuscript has been provisionally accepted, please log into Editorial Manager and update your profile, if needed. Go to http://www.editorialmanager.com/ppathogens, log in, and click on the "Update My Information" link at the top of the page. Please update your user information to ensure an efficient production and billing process.

(6) LaTeX users only: Our staff will ask you to upload a TEX file in addition to the PDF before the paper can be sent to typesetting, so please carefully review our Latex Guidelines http://journals.plos.org/plospathogens/s/latex in the meantime.

Thank you again for supporting Open Access publishing; we are looking forward to publishing your work in PLOS Pathogens.

Best regards,

Álvaro Acosta-Serrano

Academic Editor

PLOS Pathogens

Edward Mitre

Section Editor

PLOS Pathogens

Dominique Soldati-Favre

Editor-in-Chief

PLOS Pathogens

***********************************************************

Reviewer's Responses to Questions

**Part I - Summary**

Please use this section to discuss strengths/weaknesses of study, novelty/significance, general execution and scholarship.<br/><br/>

Reviewer #1: Thank you for addressing my comments

Reviewer #2: The authors addressed all my comments with satisfactory outcomes.

--------------------

**Part II – Major Issues: Key Experiments Required for Acceptance**

Please use this section to detail the key new experiments or modifications of existing experiments that should be absolutely required to validate study conclusions.<br/><br/>Generally, there should be no more than 3 such required experiments or major modifications for a "Major Revision" recommendation. If more than 3 experiments are necessary to validate the study conclusions, then you are encouraged to recommend "Reject".<br/><br/>

Reviewer #1: (No Response)

Reviewer #2: (No Response)

--------------------

**Part III – Minor Issues: Editorial and Data Presentation Modifications**

Please use this section for editorial suggestions as well as relatively minor modifications of existing data that would enhance clarity.<br/><br/>

Reviewer #1: (No Response)

Reviewer #2: (No Response)

--------------------

PLOS authors have the option to publish the peer review history of their article (what does this mean?). If published, this will include your full peer review and any attached files.

If you choose “no”, your identity will remain anonymous but your review may still be made public.

**Do you want your identity to be public for this peer review?**  For information about this choice, including consent withdrawal, please see our Privacy Policy.

Reviewer #1: Yes: Henry McSorley

Reviewer #2: No

# Editor report: Acceptance letter

**DOI:** 10.1371/journal.ppat.1014732.r004

**Authors:** Edward Mitre, Álvaro Acosta-Serrano

Dear Prof. Su,

We are delighted to inform you that your manuscript, "SR-A1 cooperates with TLR4-NF-κB signaling to drive macrophage TGF-β1 expression and Treg induction during schistosome infection," has been formally accepted for publication in PLOS Pathogens.

We have now passed your article onto the PLOS Production Department who will complete the rest of the pre-publication process. All authors will receive a confirmation email upon publication.

Thank you again for supporting open-access publishing; we are looking forward to publishing your work in PLOS Pathogens.

Best regards,

Dominique Soldati-Favre

Editor-in-Chief

PLOS Pathogens
